# Hydroxide-Mediated S_N_Ar Rearrangement for Synthesis of Novel Depside Derivatives Containing Diaryl Ether Skeleton as Antitumor Agents

**DOI:** 10.3390/molecules28114303

**Published:** 2023-05-24

**Authors:** Xiang Yu, Yinkai Xi, Yi Sui, Yang Liu, Guifen Chen, Minjie Zhang, Yan Zhang, Guoyong Luo, Yi Long, Wude Yang

**Affiliations:** 1 College of Pharmacy, Guizhou University of Traditional Chinese Medicine, Guiyang 550025, China; 2 Guizhou Joint Laboratory for International Cooperation in Ethnic Medicine, Guizhou University of Traditional Chinese Medicine, Guiyang 550025, China; 3 School of Basic Medicine, Guizhou University of Traditional Chinese Medicine, Guiyang 550025, China

**Keywords:** barbatic acid, diaryl ethers, S_N_Ar rearrangement, antitumor activity

## Abstract

A simple and efficient hydroxide-mediated S_N_Ar rearrangement was reported to synthesize new depside derivatives containing the diaryl ether skeleton from the natural product barbatic acid. The prepared compounds were determined using ^1^H NMR, ^13^C NMR, HRMS, and X-ray crystallographic analysis and were also screened in vitro for cytotoxicity against three cancer cell lines and one normal cell line. The evaluation results showed that compound **3b** possessed the best antiproliferative activity against liver cancer HepG2 cell line and low toxicity, which made it worth further study.

## 1. Introduction

**Figure 1:**
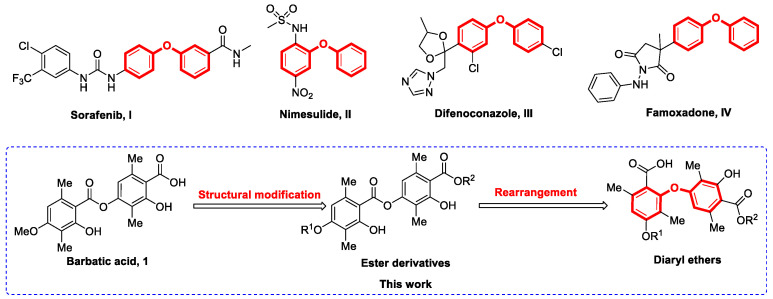
Design of novel diaryl ethers as antitumor agents.

Depsides are dimers formed by ester bonds between two aromatic rings, which are the main secondary metabolites of lichens [1]. Previous research reported that depsides had good biological properties, such as antitumor, antioxidant, and antibacterial activities [2,3,4]. Barbatic acid (**1**, Figure 1) is one kind of depside that was widely discovered from the lichen [5] and was determined to have a variety of biological activities, including anticancer, schistosomicidal, diuretic, and the potential to inhibit the growth of plants and algal [6,7,8,9]. However, to the best of our knowledge, little attention has been paid to the further structural modification of barbatic acid for developing potential antitumor agents.

Diaryl ethers are an important class of organic compounds with two aromatic rings and a flexible oxygen bridge, which are widely used in various fields such as medicine and pesticides [10]. For instance, Sorafenib (**I**, Figure 1) is a highly effective small molecule inhibitor with anticancer properties, which is used to treat advanced renal cancer [11]. Nimesulide (**II**, Figure 1), a nonsteroidal anti-inflammatory drug, has been popularized for several decades [12]. Difenoconazole (**III**, Figure 1) and famoxadone (**IV**, Figure 1) have been utilized as fungicides to protect a wide range of plants, such as rice, cotton, and cereals, from diseases [13,14]. Usually, diaryl ethers are mainly achieved through the coupling of phenols and aryl halides under the action of catalysts to form C–O bonds. These methods have certain deficiencies, such as high temperatures, expensive catalysts, or toxic solvents [15,16,17,18,19].

Herein, we reported an interesting means of converting some barbatic acid esters to novel diaryl ethers through a hydroxide-mediated S_N_Ar rearrangement reaction. The synthesized compounds were also tested for antitumor activity, and some compounds demonstrated good effects.

## 2. Results and Discussion

**Figure 2:**
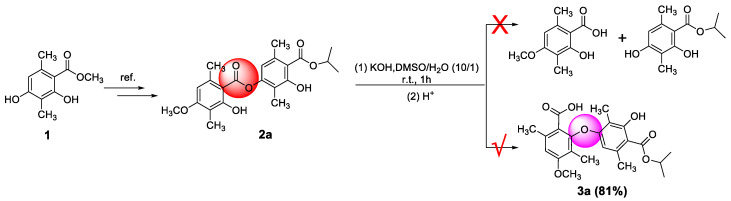
An unanticipated S_N_Ar reaction leading to **3a**.

**Figure 3:**
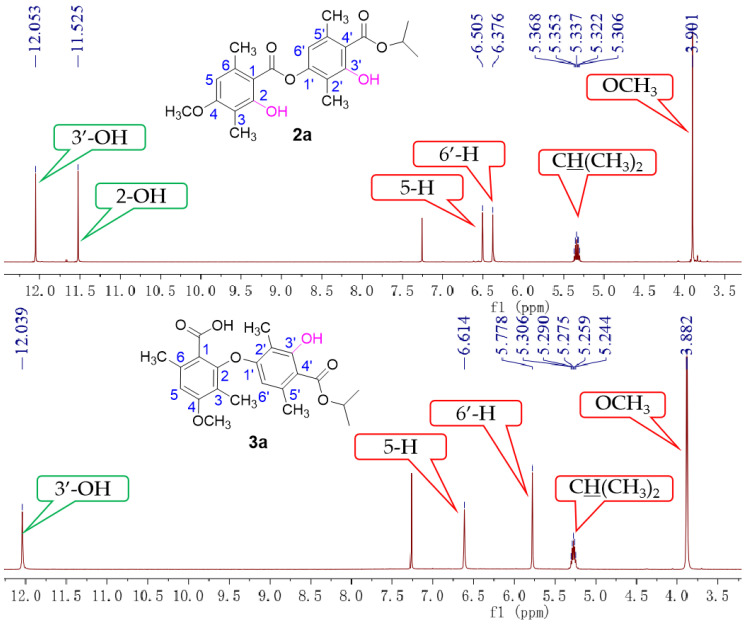
The ^1^H NMR spectra of **2a** and **3a**.

**Figure 4:**
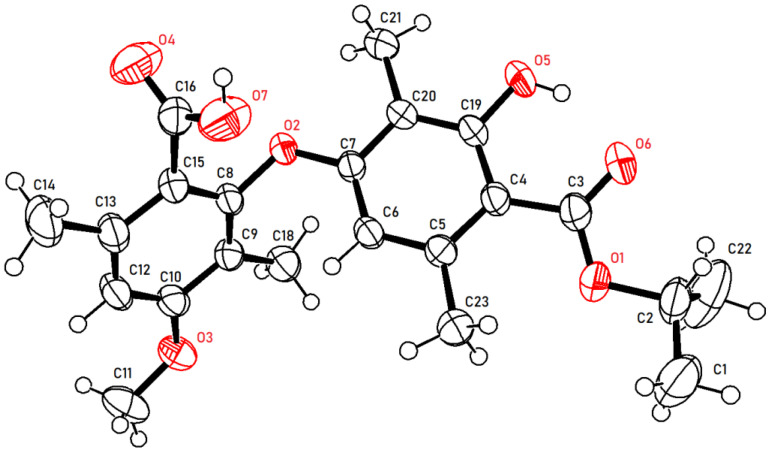
X-ray crystallographic structures of **3a**.

As illustrated in Figure 2, firstly, 3-hydroxy-4-(isopropoxycarbonyl)-2,5-dimethylphenyl 2-hydroxy-4-methoxy-3,6-dimethylbenzoate (**2a**) was prepared by our previously reported method [20]. Subsequently, **2a** reacted with potassium hydroxide in the mixed solvent (DMSO/water = 10/1, *v*/*v*) at room temperature. To our delight, the reaction did not undergo hydrolysis to produce corresponding benzoic acid and phenol as we expected but instead underwent a rearrangement reaction to produce diaryl ethers with a yield of 81% (**3a**). This was proven by comparison with the partial ^1^H NMR spectra of compounds **2a** and **3a** (Figure 3). Compared to compound **2a**, compound **3a** had only one phenolic hydroxyl group, and the chemical shift of aromatic signals was shifted from 6.37 ppm to 5.77 ppm due to the steric effects. There was no significant change in the other chemical shifts. Moreover, the X-ray crystal structure (Figure 4) of compound **3a** further demonstrated this conclusion.

**Figure 5:**
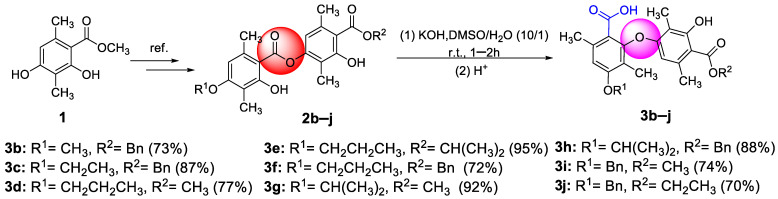
The synthetic route to compound **3b**–**j**.

**Figure 6:**
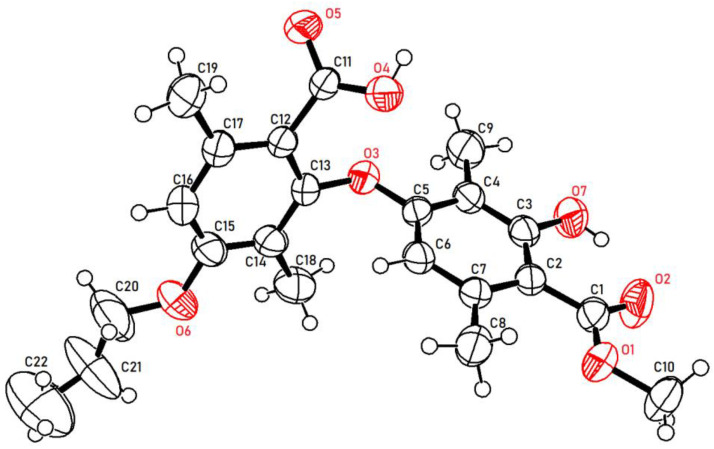
X-ray crystallographic structures of **3d**.

In order to test the reliability of the method, compounds **3b**–**j** continued to be synthesized with a 70–95% yield under the same reaction conditions, as shown in Figure 5. The structures of all target compounds were characterized using ^1^H NMR, ^13^C NMR, and HRMS. The stereochemistry of **3d** was further confirmed by X-ray crystallographic analysis (Figure 6).

**Figure 7:**
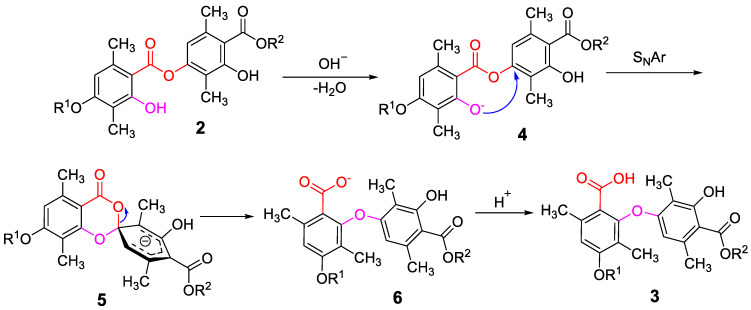
Possible mechanism for synthesis of compounds **3a**–**j**.

In addition, a probable reaction mechanism for this rearrangement was proposed and is illustrated in Figure 7. The sequence began with a simple acid-base reaction wherein hydroxide deprotonated the hydroxyl group, as shown in pink in structure **2**. The resulting phenoxide then participated in an intramolecular S_N_Ar cyclization onto the second ring, as illustrated by the conversion of structure **4** to the spirocyclic intermediate **5**. The re-establishment of aromaticity was accompanied by the formation of the carboxylate anion shown in structure **6**, and then acidification delivered the target structures **3**.

Compounds **3a**–**j** were screened for their antitumor activities against three human cancer cell lines (lung cancer A549 cells, liver cancer HepG2 cells, and prostatic cancer 22RV1 cells) in vitro by the standard MTT method, and the IC_50_ values are presented in Table 1. The experimental results showed that compounds **3a**, **3c**, **3e**, and **3f** exhibited moderate cytotoxic activities against A549 cells with IC_50_ values of 2.61, 1.43, and 2.21 mmol/L, respectively. Compounds **3a**–**d**, **i**–**j** induced high cytotoxic activity against HepG2 cells, which exhibited excellent activities with IC_50_ values of 0.41–1.56 mmol/L. Compound **3d** afforded the best antiproliferation activities toward 22RV1 cells, with an IC_50_ value of 0.78 mmol/L. The results demonstrated that diaryl ethers synthesized in this study possessed potential antitumor activities.

**Figure 8:**
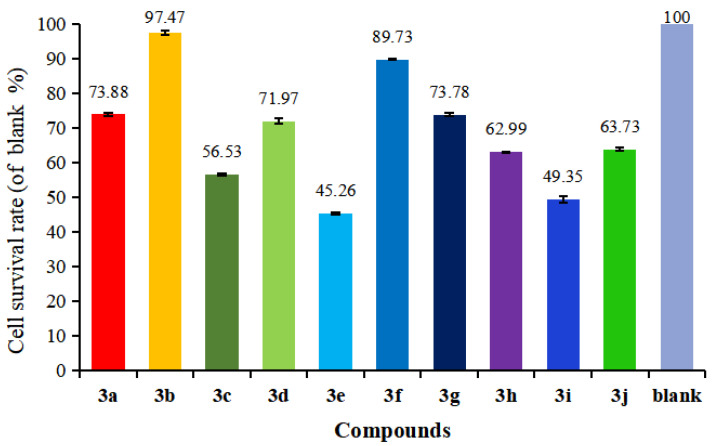
The cytotoxicity of compounds **3a**–**j** on normal MDCK Cells at 100 μmol/L.

Selective killing of cancer cells without affecting normal cell growth is an important feature that must be considered in cancer chemotherapy. Therefore, the target compounds **3a**–**j** were estimated for cytotoxicity toward normal madin-daby canine kidney cells (MDCK) and as shown in Figure 8. Compared to the blank control, most compounds could influence the growth of normal MDCK cells with cell survival rates below 90%, except for compound **3b** (97.47%), suggesting that **3b** had almost no toxicity to normal cells and could selectively inhibit the growth of liver cancer HepG2 cells.

## 3. Materials and Methods

### 3.1. Chemistry

All reagents and solvents were of reagent grade or were purified according to standard methods before use. Analytical thin-layer chromatography (TLC) was performed with silica gel plates using silica gel 60 GF_254_ (Qingdao Haiyang Chemical Co., Ltd., Qingdao, China). Melting points were determined on an XT-4 digital melting point apparatus (Beijing Tech Instrument Co., Ltd., Beijing, China) and were uncorrected. Proton nuclear magnetic resonance spectra (^1^H NMR) were recorded on a Bruker Avance DMX 400 MHz instrument (Bruker, Bremerhaven, Germany) in CDCl_3_ or DMSO-*d*_6_ using TMS (tetramethylsilane) as the internal standard. High-resolution mass spectrometry (HRMS) was carried out with a Xevo G2-SQTOF instrument (Waters, Milford, MA, USA).

#### 3.1.1. Synthesis of Intermediates **2a**–**j**

Compounds **2a**–**j** were prepared, as described in our previous publication [20], and their spectra data are shown below.

Data for *3-hydroxy-4-(isopropoxycarbonyl)-2,5-dimethylphenyl 2-hydroxy-4-methoxy-3,6-dimethylbenzoate* (**2a**): Yield: 48%, white solid, mp 135–138 °C; ^1^H NMR (400 MHz, CDCl_3_): *δ* 12.05 (s, 1H), 11.53 (s, 1H), 6.50 (s, 1H), 6.38 (s, 1H), 5.34 (p, *J* = 6.3 Hz, 1H), 3.90 (s, 3H), 2.69 (s, 3H), 2.55 (s, 3H), 2.10 (s, 3H), 2.08 (s, 3H), 1.41 (d, *J* = 6.3 Hz, 6H); ^13^C NMR (100 MHz, CDCl_3_): *δ* 171.3, 170.2, 162.9, 162.8, 162.2, 152.3, 140.7, 139.6, 116.9, 116.2, 111.3, 110.4, 106.4, 104.3, 69.8, 55.5, 25.1, 24.2, 21.9 × 2, 9.3, 7.8; HRMS *m*/*z* calcd for C_22_H_26_O_7_Na ([M + Na]^+^) 425.1570, found 425.1566.

Data for *Benzyl 2-hydroxy-4-((2-hydroxy-4-methoxy-3,6-dimethylbenzoyl)oxy)-3,6-dimethyl benzoate* (**2b**): Yield: 50%, white solid, mp 113–115 °C; ^1^H NMR (400 MHz, CDCl_3_): *δ* 11.92 (s, 1H), 11.51 (s, 1H), 7.48–7.34 (m, 5H), 6.51 (s, 1H), 6.38 (s, 1H), 5.43 (s, 2H), 3.90 (s, 3H), 2.69 (s, 3H), 2.52 (s, 3H), 2.10 (s, 3H), 2.09 (s, 3H); ^13^C NMR (100 MHz, CDCl_3_): *δ* 172.0, 170.6, 163.5 × 2, 162.7, 153.0, 141.2, 140.3, 135.5, 129.2 × 2, 129.1, 128.9 × 2, 117.5, 116.9, 111.8, 110.4, 106.9, 104.8, 67.9, 56.0, 25.5, 24.8, 9.8, 8.3; HRMS *m*/*z* calcd. for C_26_H_26_O_7_Na ([M + Na]^+^) 473.1570, found 473.1569.

Data for *benzyl 4-(4-ethoxy-2-hydroxy-3,6-dimethylbenzoyloxy)-2-hydroxy-3,6-dimethylbenzoate* (**2c**): Yield: 36%, white solid, mp 129–131 °C; ^1^H NMR (400 MHz, CDCl_3_): *δ* 11.93 (s, 1H), 11.52 (s, 1H), 7.47–7.33 (m, 5H), 6.51 (s, 1H), 6.36 (s, 1H), 5.43 (s, 2H), 4.12 (q, *J* = 6.8 Hz, 2H), 2.67 (s, 3H), 2.53 (s, 3H), 2.11 (s, 3H), 2.10 (s, 3H), 1.46 (t, *J* = 6.8 Hz, 3H); ^13^C NMR (100 MHz, CDCl_3_): *δ* 171.6, 170.2, 163.1, 163.0, 161.8, 152.6, 140.6, 139.8, 135.1, 128.7 × 2, 128.6, 128.5 × 2, 117.0, 116.4, 111.3, 109.8, 107.3, 104.1, 67.5, 63.8, 25.1, 24.4, 14.8, 9.3, 7.9; HRMS *m*/*z* calcd for C_27_H_28_O_7_Na ([M + Na]^+^) 487.1727, found 487.1729.

Data for *3-hydroxy-4-(methoxycarbonyl)-2,5-dimethylphenyl 2-hydroxy-3,6-dimethyl-4-propoxybenzoate* (**2d**): Yield: 33%, white solid, mp 110–113 °C; ^1^H NMR (400 MHz, CDCl_3_): *δ* 11.91 (s, 1H), 11.50 (s, 1H), 6.51 (s, 1H), 6.34 (s, 1H), 4.00 (t, *J* = 6.4 Hz, 2H), 3.96 (s, 3H), 2.65 (s, 3H), 2.52 (s, 3H), 2.10 (s, 3H), 2.07 (s, 3H), 1.85 (p, *J* = 6.9 Hz, 2H), 1.06 (t, *J* = 7.4 Hz, 3H); ^13^C NMR (100 MHz, CDCl_3_): δ 172.3, 170.2, 163.0, 162.8, 161.9, 152.5, 140.6, 139.6, 116.9, 116.4, 111.4, 109.9, 107.3, 104.0, 69.7, 52.2, 25.0, 24.0, 22.6, 10.5, 9.3, 7.8; HRMS *m*/*z* calcd for C_22_H_26_O_7_Na ([M + Na]^+^) 425.1570, found 425.1564.

Data for *3-hydroxy-4-(isopropoxycarbonyl)-2,5-dimethylphenyl 2-hydroxy-3,6-dimethyl-4-propoxybenzoate* (**2e**): Yield: 37%, white solid, mp 112–115 °C; ^1^H NMR (400 MHz, CDCl_3_): *δ* 12.05 (s, 1H), 11.53 (s, 1H), 6.50 (s, 1H), 6.36 (s, 1H), 5.38-5.29 (m, 1H), 4.01 (t, *J* = 6.4 Hz, 2H), 2.67 (s, 3H), 2.54 (s, 3H), 2.11 (s, 3H), 2.08 (s, 3H), 1.89–1.80 (m, 2H), 1.42 (s, 3H), 1.41 (d, *J* = 6.3 Hz, 6H), 1.07 (t, *J* = 7.2 Hz, 3H); ^13^C NMR (100 MHz, CDCl_3_): *δ* 171.3, 170.3, 163.0, 162.8, 161.9, 152.3, 140.6, 139.6, 116.9, 116.3, 111.4, 110.4, 107.3, 104.1, 69.8, 69.7, 25.0, 24.2, 22.6, 21.9 × 2, 10.5, 9.3, 7.8; HRMS *m*/*z* calcd for C_24_H_30_O_7_Na ([M + Na]^+^) 453.1883, found 453.1883.

Data for *benzyl 2-hydroxy-4-(2-hydroxy-3,6-dimethyl-4-propoxybenzoyloxy)-3,6-dimethylbenzoate* (**2f**): Yield: 30%, white solid, mp 111–114 °C; ^1^H NMR (400 MHz, CDCl_3_): *δ* 11.92 (s, 1H), 11.51 (s, 1H), 7.51–7.29 (m, 5H), 6.51 (s, 1H), 6.36 (s, 1H), 5.43 (s, 2H), 4.02 (t, *J* = 6.4 Hz, 2H), 2.67 (s, 3H), 2.52 (s, 3H), 2.11 (s, 3H), 2.09 (s, 3H), 1.86 (q, *J* = 6.5 Hz, 2H), 1.07 (t, *J* = 7.4 Hz, 3H); ^13^C NMR (100 MHz, CDCl_3_): *δ* 171.6, 170.2, 163.0, 163.0, 161.9, 152.6, 140.6, 139.7, 135.1, 128.7 × 2, 128.6, 128.4 × 2, 117.0, 116.4, 111.4, 109.9, 107.3, 104.1, 69.7, 67.4, 25.0, 24.3, 22.6, 10.5, 9.3, 7.8; HRMS *m*/*z* calcd for C_28_H_30_O_7_Na ([M + Na]^+^) 501.1883, found 501.1880.

Data for *3-hydroxy-4-(methoxycarbonyl)-2,5-dimethylphenyl 2-hydroxy-4-isopropoxy-3,6-dimethylbenzoate* (**2g**): Yield: 36%, white solid, mp 60–63 °C; ^1^H NMR (400 MHz, CDCl_3_): *δ* 11.92 (s, 1H), 11.53 (s, 1H), 6.51 (s, 1H), 6.37 (s, 1H), 4.67 (p, *J* = 6.1 Hz, 1H), 3.97 (s, 3H), 2.66 (s, 3H), 2.53 (s, 3H), 2.09 (s, 6H), 1.38 (d, *J* = 6.0 Hz, 6H); ^13^C NMR (100 MHz, CDCl_3_): *δ* 172.3, 170.2, 163.4, 162.8, 161.0, 152.6, 140.3, 139.6, 116.9, 116.4, 112.3, 109.9, 108.6, 103.8, 70.3, 52.2, 25.1, 24.0, 22.2 × 2, 9.3, 8.0; HRMS *m*/*z* calcd for C_22_H_26_O_7_Na ([M + Na]^+^) 425.1570, found 425.1568.

Data for *benzyl 2-hydroxy-4-(2-hydroxy-4-isopropoxy-3,6-dimethylbenzoyloxy)-3,6-dimethylbenzoate* (**2h**): Yield: 40%, white solid, mp 83–86 °C; ^1^H NMR (400 MHz, CDCl_3_): *δ* 11.91 (s, 1H), 11.53 (s, 1H), 7.47–7.33 (m, 5H), 6.50 (s, 1H), 6.36 (s, 1H), 5.42 (s, 2H), 4.66 (p, *J* = 6.1 Hz, 1H), 2.65 (s, 3H), 2.51 (s, 3H), 2.08 (s, 6H), 1.37 (d, *J* = 6.0 Hz, 6H); ^13^C NMR (100 MHz, CDCl_3_): *δ* 171.6, 170.2, 163.4, 163.0, 161.0, 152.6, 140.3, 139.7, 135.1, 128.7 × 2, 128.6, 128.4 × 2, 117.0, 116.4, 112.3, 109.8, 108.6, 103.8, 70.3, 67.5, 25.1, 24.3, 22.2 × 2, 9.3, 8.0; HRMS *m*/*z* calcd for C_28_H_30_O_7_Na ([M + Na]^+^) 501.1883, found 501.1883.

Data for *3-hydroxy-4-(methoxycarbonyl)-2,5-dimethylphenyl 4-(benzyloxy)-2-hydroxy-3,6-dimethylbenzoate* (**2i**): Yield: 35%, white solid, mp 131–134 °C; ^1^H NMR (400 MHz, CDCl_3_): *δ* 11.93 (s, 1H), 11.54 (s, 1H), 7.48–7.33 (m, 5H), 6.52 (s, 1H), 6.45 (s, 1H), 5.18 (s, 2H), 3.98 (s, 3H), 2.67 (s, 3H), 2.54 (s, 3H), 2.18 (s, 3H), 2.09 (s, 3H); ^13^C NMR (100 MHz, CDCl_3_): *δ* 172.7, 170.6, 163.6, 163.3, 161.8, 152.9, 141.1, 140.1, 137.1, 129.1 × 2, 128.4, 127.5 × 2, 117.3, 116.8, 112.2, 110.4, 108.1, 105.0, 70.4, 52.7, 25.5, 24.4, 9.7, 8.5; HRMS *m*/*z* calcd for C_26_H_26_O_7_Na ([M + Na]^+^) 473.1570, found 473.1569.

Data for *4-(ethoxycarbonyl)-3-hydroxy-2,5-dimethylphenyl 4-(benzyloxy)-2-hydroxy-3,6-dimethylbenzoate* (**2j**): Yield: 40%, white solid, mp 123–126 °C; ^1^H NMR (400 MHz, CDCl_3_): *δ* 12.00 (s, 1H), 11.54 (s, 1H), 7.47–7.32 (m, 5H), 6.51 (s, 1H), 6.44 (s, 1H), 5.17 (s, 2H), 4.45 (q, *J* = 7.1 Hz, 2H), 2.67 (s, 3H), 2.55 (s, 3H), 2.17 (s, 3H), 2.08 (s, 3H), 1.43 (t, *J* = 7.1 Hz, 3H); ^13^C NMR (100 MHz, CDCl_3_): *δ* 171.8, 170.2, 163.1, 162.8, 161.4, 152.4, 140.6, 139.7, 136.6, 128.6 × 2, 128.0, 127.0 × 2, 116.9, 116.3, 111.8, 110.1, 107.6, 104.5, 69.9, 61.7, 25.1, 24.1, 14.2, 9.3, 8.1; HRMS *m*/*z* calcd for C_27_H_28_O_7_Na ([M + Na]^+^) 487.1727, found 487.1721.

#### 3.1.2. Synthesis of Target Compounds **3a**–**j**

To a stirred solution of potassium hydroxide (67.3 mg, 1.2 mmol) in a mixed solvent (20 mL, DMSO/water = 10/1, *v*/*v*) at room temperature, **2a**–**j** (0.6 mmol) was added. The reaction mixture was stirred for 1–2 h. Subsequently, the pH of the reaction mixture was adjusted to 1–2 with 1 mol/L hydrochloric acid, and the crude solid was collected by filtration before it was recrystallized with ethanol to afford **3a**–**j** in 70–95%.

Data for *2-(3-hydroxy-4-(isopropoxycarbonyl)-2,5-dimethylphenoxy)-4-methoxy-3,6-dimethylbenzoic acid* (**3a**): Yield: 81%, white solid, mp 206–208 °C; ^1^H NMR (400 MHz, CDCl_3_) *δ* 12.04 (s, 1H), 6.61 (s, 1H), 5.78 (s, 1H), 5.27 (p, *J* = 6.3 Hz, 1H), 3.88 (s, 3H), 2.45 (s, 3H), 2.35 (s, 3H), 2.23 (s, 3H), 1.89 (s, 3H), 1.37 (s, 3H), 1.36 (s, 3H); ^13^C NMR (100 MHz, CDCl_3_) *δ* 171.6, 162.6, 159.7, 159.6, 150.9, 139.8, 137.0, 134.6, 117.8, 111.1, 109.4, 108.3, 106.5 × 2, 69.1, 55.7, 24.6, 22.0 × 2, 20.6, 8.9, 8.1; HRMS *m*/*z* calcd for C_22_H_26_O_7_Na ([M + Na]^+^) 425.1571, found 425.1569 (Figures S1–S3).

Data for *2-(4-(benzyloxycarbonyl)-3-hydroxy-2,5-dimethylphenoxy)-4-methoxy-3,6-dimethylbenzoic acid* (**3b**): Yield: 73%, white solid, mp 186–188 °C; ^1^H NMR (400 MHz, CDCl_3_) *δ* 11.90 (s, 1H), 7.43–7.30 (m, 5H), 6.60 (s, 1H), 5.78 (s, 1H), 5.36 (s, 2H), 3.87 (s, 3H), 2.44 (s, 3H), 2.32 (s, 3H), 2.22 (s, 3H), 1.88 (s, 3H); ^13^C NMR (100 MHz, CDCl_3_) *δ* 171.8, 169.9, 162.8, 160.0, 159.6, 150.8, 139.9, 136.9, 135.4, 128.6 × 2, 128.4 × 2, 128.3, 119.6, 117.7, 111.2, 109.4, 108.4, 106.0, 66.9, 55.7, 24.7, 20.6, 8.9, 8.1; HRMS *m*/*z* calcd for C_26_H_26_O_7_Na ([M + Na]^+^) 473.1570, found 473.1567 (Figures S4–S6).

Data for *2-(4-(benzyloxycarbonyl)-3-hydroxy-2,5-dimethylphenoxy)-4-ethoxy-3,6-dimethylbenzoic acid* (**3c**): Yield: 87%, white solid, mp 190–192 °C; ^1^H NMR (400 MHz, CDCl_3_) *δ* 11.90 (s, 1H), 7.45–7.27 (m, 5H), 6.59 (s, 1H), 5.75 (s, 1H), 5.36 (s, 2H), 4.08 (q, *J* = 6.9 Hz, 2H), 2.43 (s, 3H), 2.32 (s, 3H), 2.21 (s, 3H), 1.88 (s, 3H), 1.45 (t, *J* = 6.9 Hz, 3H). ^13^C NMR (100 MHz, CDCl_3_) *δ* 171.8, 170.7, 162.9, 159.9, 159.5, 151.3, 139.9, 137.6, 135.5, 128.6 × 2, 128.4 × 2, 128.3, 118.2, 118.1, 111.3, 110.5, 108.3, 106.1, 66.9, 64.0, 24.7, 20.9, 14.7, 9.0, 8.0; HRMS *m*/*z* calcd for C_27_H_28_O_7_Na ([M + Na]^+^) 487.1727, found 487.1729 (Figures S7–S9).

Data for *2-(3-hydroxy-4-(methoxycarbonyl)-2,5-dimethylphenoxy)-3,6-dimethyl-4-propoxybenzoic acid* (**3d**): Yield: 77%, white solid, mp 234–236 °C; ^1^H NMR (400 MHz, CDCl_3_) *δ* 11.90 (s, 1H), 6.60 (s, 1H), 5.80 (s, 1H), 3.98 (t, *J* = 6.4 Hz, 2H), 3.90 (s, 3H), 2.44 (s, 3H), 2.34 (s, 3H), 2.23 (s, 3H), 1.90 (s, 3H), 1.84 (p, *J* = 7.1 Hz, 2H), 1.06 (t, *J* = 7.4 Hz, 3H). ^13^C NMR (100 MHz, CDCl_3_) *δ* 172.5, 170.0, 162.6, 159.9, 159.3, 151.0, 139.8, 137.2, 118.9, 118.0, 111.1, 110.4, 108.4, 106.1, 69.9, 51.8, 24.4, 22.6, 20.7, 10.6, 8.9, 8.1; HRMS *m*/*z* calcd for C_22_H_26_O_7_Na ([M + Na]^+^) 425.1571, found 425.1566 (Figures S10–S12).

Data for *2-(3-hydroxy-4-(isopropoxycarbonyl)-2,5-dimethylphenoxy)-3,6-dimethyl-4-propoxybenzoic acid* (**3e**): Yield: 95%, white solid, mp 226–228 °C; ^1^H NMR (400 MHz, CDCl_3_) *δ* 12.05 (s, 1H), 6.60 (s, 1H), 5.76 (s, 1H), 5.33–5.22 (m, 1H), 3.99 (t, *J* = 6.6 Hz, 2H), 2.45 (s, 3H), 2.35 (s, 3H), 2.21 (s, 3H), 1.89 (s, 3H), 1.84 (dt, *J* = 13.9, 7.0 Hz, 2H), 1.37 (s, 3H), 1.36 (s, 3H), 1.06 (t, *J* = 7.4 Hz, 3H); ^13^C NMR (100 MHz, CDCl_3_) *δ* 171.6, 170.9, 162.7, 159.6, 159.6, 151.3, 139.8, 137.6, 118.2, 118.1, 111.2, 110.4, 108.2, 106.6, 69.9, 69.1, 24.6, 22.6, 22.0 × 2, 20.9, 10.5, 8.9, 8.0; HRMS *m*/*z* calcd for C_24_H_30_O_7_Na ([M + Na]^+^) 453.1883, found 453.1878 (Figures S13–S15).

Data for *2-(4-(benzyloxycarbonyl)-3-hydroxy-2,5-dimethylphenoxy)-3,6-dimethyl-4-propoxybenzoic acid* (**3f**): Yield: 72%, white solid, mp 176–178 °C; ^1^H NMR (400 MHz, CDCl_3_) *δ* 11.90 (s, 1H), 7.43-7.29 (m, 5H), 6.60 (s, 1H), 5.76 (s, 1H), 5.36 (s, 2H), 3.98 (t, *J* = 6.5 Hz, 2H), 2.43 (s, 3H), 2.32 (s, 3H), 2.22 (s, 3H), 1.89 (s, 3H), 1.84 (dt, *J* = 13.9, 7.0 Hz, 2H), 1.06 (t, *J* = 7.4 Hz, 3H); ^13^C NMR (100 MHz, CDCl_3_) *δ* 171.8, 162.8, 160.0, 159.9, 159.6, 151.2, 139.9, 137.6, 135.4, 128.6 × 2, 128.4 × 2, 128.3, 118.1, 111.3, 110.5, 108.3, 106.1, 106.1, 69.9, 66.9, 24.7, 22.6, 20.9, 10.5, 8.9, 8.1; HRMS *m*/*z* calcd for C_28_H_30_O_7_Na ([M + Na]^+^) 501.1884, found 501.1878 (Figures S16–S18).

Data for *2-(3-hydroxy-4-(methoxycarbonyl)-2,5-dimethylphenoxy)-4-isopropoxy-3,6-dimethylbenzoic acid* (**3g**): Yield: 92%, white solid, mp 232–234 °C; ^1^H NMR (400 MHz, CDCl_3_) *δ* 11.90 (s, 1H), 6.63 (s, 1H), 5.78 (s, 1H), 4.62 (hept, *J* = 6.0 Hz, 1H), 3.90 (s, 3H), 2.43 (s, 3H), 2.34 (s, 3H), 2.21 (s, 3H), 1.87 (s, 3H), 1.37 (s, 6H); ^13^C NMR (100 MHz, CDCl_3_) *δ* 172.5, 171.1, 162.7, 159.8, 158.6, 151.5, 139.8, 137.3, 119.1, 118.2, 112.1, 111.2, 108.3, 106.2, 70.8, 51.8, 24.4 × 2, 22.1, 20.9, 9.2, 8.0; HRMS *m*/*z* calcd for C_22_H_26_O_7_Na ([M + Na]^+^) 425.1571, found 425.1563 (Figures S19–S21).

Data for *2-(4-(benzyloxycarbonyl)-3-hydroxy-2,5-dimethylphenoxy)-4-isopropoxy-3,6-dimethylbenzoic acid* (**3h**): Yield: 88%, white solid, mp 156–158 °C; ^1^H NMR (400 MHz, CDCl_3_) *δ* 11.90 (s, 1H), 7.44–7.29 (m, 5H), 6.62 (s, 1H), 5.76 (s, 1H), 5.36 (s, 2H), 4.61 (hept, *J* = 6.1 Hz, 1H), 2.43 (s, 3H), 2.32 (s, 3H), 2.21 (s, 3H), 1.86 (s, 3H), 1.40-1.33 (m, 6H); ^13^C NMR (100 MHz, CDCl_3_) *δ* 171.8, 170.6, 162.8, 159.9, 158.6, 151.5, 139.9, 137.4, 135.4, 128.6 × 2, 128.4 × 2, 128.3, 119.1, 118.1, 112.2, 111.3, 108.3, 106.1, 70.8, 66.9, 24.7 × 2, 22.1, 20.9, 9.2, 8.0; HRMS *m*/*z* calcd for C_28_H_30_O_7_Na ([M + Na]^+^) 501.1883, found 501.1884 (Figures S22–S24).

Data for *4-(benzyloxy)-2-(3-hydroxy-4-(methoxycarbonyl)-2,5-dimethylphenoxy)-3,6-dimethylbenzoic acid* (**3i**): Yield: 74%, white solid, mp 223–225 °C; ^1^H NMR (400 MHz, CDCl_3_) *δ* 11.91 (s, 1H), 7.48–7.31 (m, 5H), 6.70 (s, 1H), 5.80 (s, 1H), 5.13 (s, 2H), 3.90 (s, 3H), 2.44 (s, 3H), 2.34 (s, 3H), 2.23 (s, 3H), 1.95 (s, 3H); ^13^C NMR (100 MHz, CDCl_3_) *δ* 172.5, 169.9, 162.6, 159.9, 158.8, 151.0, 139.9, 137.0, 136.6, 128.6 × 2, 128.0, 127.2 × 2, 119.7, 118.3, 111.1, 110.8, 108.4, 106.2, 70.3, 51.8, 24.4, 20.6, 9.2, 8.1; HRMS *m*/*z* calcd for C_26_H_26_O_7_Na ([M + Na]^+^) 473.1571, found 473.1564 (Figures S25–S27).

Data for *4-(benzyloxy)-2-(4-(ethoxycarbonyl)-3-hydroxy-2,5-dimethylphenoxy)-3,6-dimethylbenzoic acid* (**3j**): Yield: 70%, white solid, mp 184-186 ^o^C; ^1^H NMR (400 MHz, CDCl_3_) δ 11.99 (s, 1H), 7.50–7.31 (m, 5H), 6.71 (s, 1H), 5.78 (s, 1H), 5.14 (s, 2H), 4.38 (q, *J* = 7.1 Hz, 2H), 2.44 (s, 3H), 2.36 (s, 3H), 2.22 (s, 3H), 1.95 (s, 3H), 1.38 (t, *J* = 7.1 Hz, 3H); ^13^C NMR (100 MHz, CDCl_3_) *δ* 172.1, 170.4, 162.7, 159.7, 159.0, 151.2, 139.9, 137.4, 136.5, 128.6 × 2, 128.1, 127.2 × 2, 119.2, 118.4, 111.2, 110.9, 108.3, 106.4, 70.3, 61.2, 24.5, 20.8, 14.2, 9.2, 8.1; HRMS *m*/*z* calcd for C_27_H_28_O_7_Na ([M + Na]^+^) 487.1727, found 487.1725 (Figures S28–S30).

### 3.2. In Vitro Cytotoxicity Assay

The cytotoxicity of test compounds against three different tumor cell lines (A549 cells, HepG2 cells, and 22RV1 cells) and one normal cell line (madin-daby canine kidney cells, DMCK) were evaluated using an MTT assay in vitro [21]. Logarithmic growth phase cells were seeded in a 96-well plate (10^4^ cells/well) and incubated in a cell culture incubator for 24 h. The culture medium was then removed and replaced with a drug-containing medium for the treatment groups (**3a**–**j**, 100 μmol/L) and normal saline for the control group. Each well was treated with 100 µL of the respective solution and incubated for an additional 24 h. The culture medium was then removed and replaced with a DMEM solution containing 20 µL of MTT (5 mg/mL). After incubating for 4 h, the liquid in each well was removed and replaced with 150 µL of DMSO, which was shaken for 10 min to ensure thorough mixing. The absorbance at 490 nm was measured to determine the optical density (OD) of each well. Cell growth inhibition rate (%) = (OD_Blank_ − OD_Experimental_)/OD_Blank_ × 100%. Cell survival rate (%) = OD_Experimental_/OD_Blank_ × 100%. Subsequently, the inhibition rates versus seven concentrations of **3a**–**j** against A549 cells, HepG2 cells, and 22RV1 cells were obtained, and then IC_50_ values were calculated according to the Logit method.

## 4. Conclusions

In summary, we have described an interesting S_N_Ar rearrangement to convert barbatic acid ester derivatives to novel diaryl ethers. The products were characterized by ^1^H NMR, ^13^C NMR, HRMS, and X-ray crystallographic analysis. In vitro, anticancer activity tests on a panel of three human cancer cell lines and one normal cell line using an MTT assay revealed that the compound **3b** had excellent activity for HepG2 cell lines and low toxicity, which could be used as a valuable lead compound for further study.

## Figures and Tables

**Table 1:** IC_50_ values (mmol/L) of compounds **3a**–**j** against three human cancer cell lines.

Compound	A549	HepG2	22RV1
**3a**	2.61	0.48	32.84
**3b**	/ ^a^	0.82	/
**3c**	21.52	0.56	/
**3d**	/	1.26	0.78
**3e**	1.43	/	/
**3f**	2.21	/	6.16
**3g**	14.83	/	/
**3h**	26.94	/	3.24
**3i**	/	0.41	/
**3j**	/	1.56	/

^a^ No inhibition action.

## Data Availability

Data are included within the manuscript or the Supporting Information File.

## Supplementary Materials

The following supporting information can be downloaded at: https://www.mdpi.com/article/10.3390/molecules28114303/s1. Figures S1–S30: ^1^H NMR, ^13^C NMR and HRMS spectrum of the compounds **3a**–**j**.
