# Supplementary material for: Hydroxide-Mediated SNAr Rearrangement for Synthesis of Novel Depside Derivatives Containing Diaryl Ether Skeleton as Antitumor Agents
Source: Molecules. 2023 May 24;28(11):4303. doi: 10.3390/molecules28114303 (PMC10254537; doi:10.3390/molecules28114303)

checkCIF/PLATON report

You have not supplied any structure factors. As a result the full set of tests cannot be run.

THIS REPORT IS FOR GUIDANCE ONLY. IF USED AS PART OF A REVIEW PROCEDURE FOR PUBLICATION, IT SHOULD NOT REPLACE THE EXPERTISE OF AN EXPERIENCED CRYSTALLOGRAPHIC REFEREE.

No syntax errors found. CIF dictionary Interpreting this report

Datablock: a

Bond precision: C-C = 0.0026 A Wavelength=0.71073

Cell:	a=12.3291(11)	b=7.9656(8)	c=25.755(2)
	alpha=90	beta=100.544(3)	gamma=90
Temperature:	273 K		

	Calculated	Reported
Volume	2486.7(4)	2486.7(4)
Space group	P 21/c	P 1 21/c 1
Hall group	-P 2ybc	-P 2ybc
Moiety formula	C22 H26 O7, C2 H6 O S	C22 H26 O7, C2 H6 O S
Sum formula	C24 H32 O8 S	C24 H32 O8 S
Mr	480.56	480.56
Dx,g cm-3	1.284	1.284
Z	4	4
Mu (mm-1)	0.175	0.175
F000	1024.0	1024.0
F000’	1025.05	
h,k,lmax	17,11,36	17,11,36
Nref	7299	7262
Tmin,Tmax		
Tmin’		

Correction method= Not given

Data completeness= 0.995 Theta(max)= 30.050

R(reflections)= 0.0601(4644) wR2(reflections)= 0.1726(7262)

S = 1.030 Npar= 317

The following ALERTS were generated. Each ALERT has the format
test-name_ALERT_alert-type_alert-level.
Click on the hyperlinks for more details of the test.

Alert level C

```
PLAT053_ALERT_1_C Minimum Crystal Dimension Missing (or Error) ...     Please Check
PLAT054_ALERT_1_C Medium  Crystal Dimension Missing (or Error) ...     Please Check
PLAT055_ALERT_1_C Maximum Crystal Dimension Missing (or Error) ...     Please Check
PLAT220_ALERT_2_C NonSolvent   Resd 1  C   Ueq(max)/Ueq(min) Range        3.5 Ratio
PLAT242_ALERT_2_C Low    'MainMol' Ueq as Compared to Neighbors of         C2 Check
PLAT242_ALERT_2_C Low    'MainMol' Ueq as Compared to Neighbors of        C16 Check
PLAT243_ALERT_4_C High   'Solvent' Ueq as Compared to Neighbors of        C24 Check
PLAT243_ALERT_4_C High   'Solvent' Ueq as Compared to Neighbors of        C27 Check
PLAT482_ALERT_4_C Small D-H..A Angle Rep for O5       ..O5       .      98.10 Degree
```

Alert level G

```
PLAT005_ALERT_5_G No Embedded Refinement Details Found  in the CIF     Please Do !
PLAT007_ALERT_5_G Number of Unrefined Donor-H Atoms .............          2 Report
PLAT093_ALERT_1_G No s.u.'s on H-positions, Refinement Reported as      mixed Check
PLAT199_ALERT_1_G Reported _cell_measurement_temperature ..... (K)        273 Check
PLAT200_ALERT_1_G Reported   _diffrn_ambient_temperature ..... (K)        273 Check
PLAT302_ALERT_4_G Anion/Solvent/Minor-Residue Disorder (Resd  2  )        25% Note
PLAT480_ALERT_4_G Long H...A H-Bond Reported H5       ..O5       .       2.66 Ang.
PLAT480_ALERT_4_G Long H...A H-Bond Reported H22A     ..O3       .       2.93 Ang.
PLAT779_ALERT_4_G Suspect or Irrelevant (Bond) Angle(s) in CIF ...      20.70 Deg.
              H24D -C24  -H24C    1_555   1_555   1_555 ......     #  119 Check
PLAT779_ALERT_4_G Suspect or Irrelevant (Bond) Angle(s) in CIF ...      35.60 Deg.
              H27D -C27  -H27C    1_555   1_555   1_555 ......     #  148 Check
PLAT779_ALERT_4_G Suspect or Irrelevant (Bond) Angle(s) in CIF ...      35.80 Deg.
              H27E -C27  -H27B    1_555   1_555   1_555 ......     #  151 Check
```

0 **ALERT level A** = Most likely a serious problem - resolve or explain
0 **ALERT level B** = A potentially serious problem, consider carefully
9 **ALERT level C** = Check. Ensure it is not caused by an omission or oversight
11 **ALERT level G** = General information/check it is not something unexpected

6 ALERT type 1 CIF construction/syntax error, inconsistent or missing data
3 ALERT type 2 Indicator that the structure model may be wrong or deficient
0 ALERT type 3 Indicator that the structure quality may be low
9 ALERT type 4 Improvement, methodology, query or suggestion
2 ALERT type 5 Informative message, check

It is advisable to attempt to resolve as many as possible of the alerts in all categories. Often the minor alerts point to easily fixed oversights, errors and omissions in your CIF or refinement strategy, so attention to these fine details can be worthwhile. In order to resolve some of the more serious problems it may be necessary to carry out additional measurements or structure refinements. However, the purpose of your study may justify the reported deviations and the more serious of these should normally be commented upon in the discussion or experimental section of a paper or in the "special_details" fields of the CIF. checkCIF was carefully designed to identify outliers and unusual parameters, but every test has its limitations and alerts that are not important in a particular case may appear. Conversely, the absence of alerts does not guarantee there are no aspects of the results needing attention. It is up to the individual to critically assess their own results and, if necessary, seek expert advice.

Publication of your CIF in IUCr journals

A basic structural check has been run on your CIF. These basic checks will be run on all CIFs submitted for publication in IUCr journals (*Acta Crystallographica*, *Journal of Applied Crystallography*, *Journal of Synchrotron Radiation*); however, if you intend to submit to *Acta Crystallographica Section C* or *E* or *IUCrData*, you should make sure that full publication checks are run on the final version of your CIF prior to submission.

Publication of your CIF in other journals

Please refer to the *Notes for Authors* of the relevant journal for any special instructions relating to CIF submission.

PLATON version of 28/11/2022; check.def file version of 28/11/2022

Datablock a - ellipsoid plot

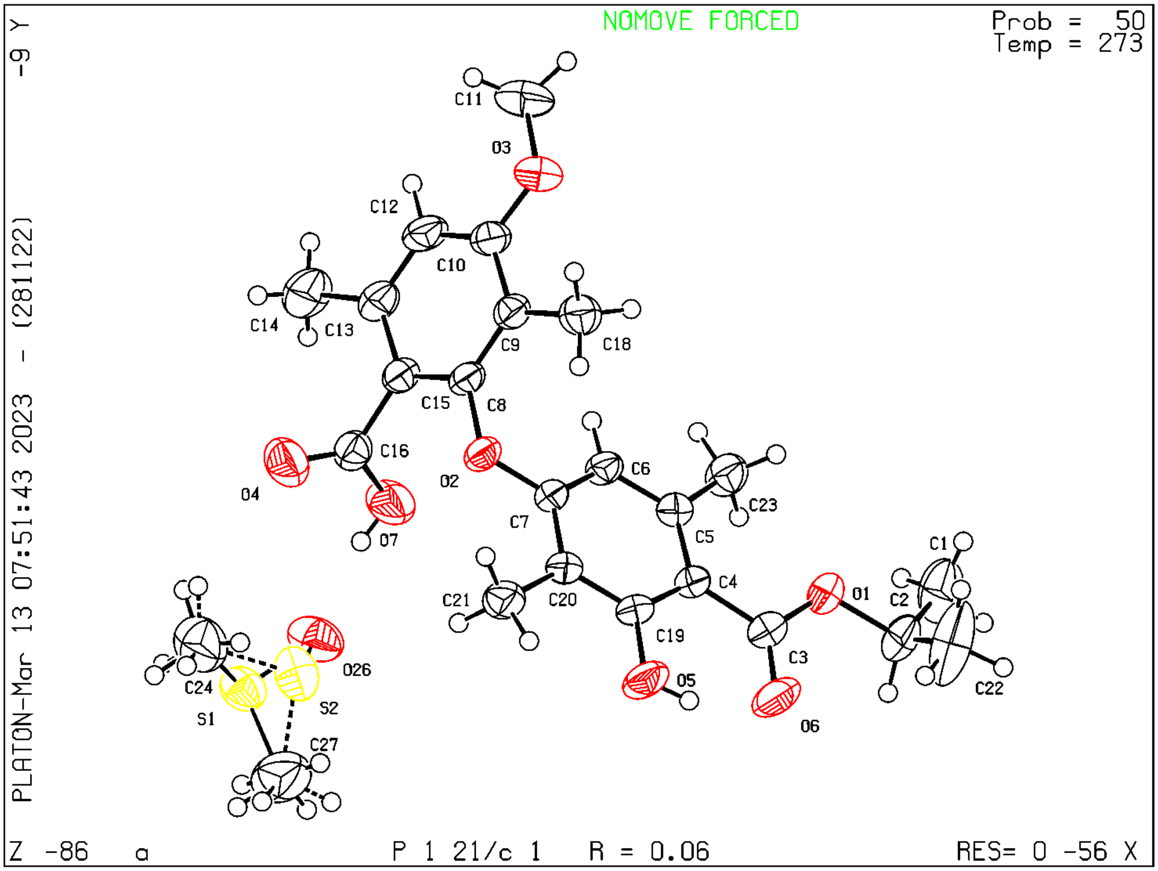

Supplement: Supplementary file 1 [file molecules-28-04303-s001.zip › checkcif (3a).pdf]
