# Supplementary material for: Hydroxide-Mediated SNAr Rearrangement for Synthesis of Novel Depside Derivatives Containing Diaryl Ether Skeleton as Antitumor Agents
Source: Molecules. 2023 May 24;28(11):4303. doi: 10.3390/molecules28114303 (PMC10254537; doi:10.3390/molecules28114303)

checkCIF/PLATON report

You have not supplied any structure factors. As a result the full set of tests cannot be run.

THIS REPORT IS FOR GUIDANCE ONLY. IF USED AS PART OF A REVIEW PROCEDURE FOR PUBLICATION, IT SHOULD NOT REPLACE THE EXPERTISE OF AN EXPERIENCED CRYSTALLOGRAPHIC REFEREE.

No syntax errors found.   CIF dictionary   Interpreting this report

Datablock: a

```
Bond precision:   C-C = 0.0047 A          Wavelength=0.71073

Cell:           a=7.6363(16)       b=11.878(3)       c=13.501(3)
                alpha=107.789(6)   beta=103.545(6)   gamma=102.577(6)
Temperature:    273 K

                 Calculated                  Reported
Volume           1076.8(4)                   1076.9(4)
Space group      P -1                        P -1
Hall group       -P 1                        -P 1
Moiety formula   C22 H26 O7                  C22 H26 O7
Sum formula      C22 H26 O7                  C22 H26 O7
Mr               402.43                      402.43
Dx,g cm-3        1.241                       1.241
Z                2                           2
Mu (mm-1)        0.092                       0.092
F000             428.0                       428.0
F000'            428.25
h,k,lmax         10,15,18                    10,15,18
Nref             5393                        5337
Tmin,Tmax
Tmin'

Correction method= Not given

Data completeness= 0.990          Theta(max)= 28.370

R(reflections)= 0.0789( 2491)                         wR2(reflections)=
                                                      0.2722( 5337)
S = 1.027                  Npar= 290
```

The following ALERTS were generated. Each ALERT has the format
test-name_ALERT_alert-type_alert-level.
Click on the hyperlinks for more details of the test.

Alert level C

```
PLAT026_ALERT_3_C Ratio Observed / Unique Reflections (too) Low ..        47% Check
PLAT053_ALERT_1_C Minimum Crystal Dimension Missing (or Error) ...     Please Check
PLAT054_ALERT_1_C Medium  Crystal Dimension Missing (or Error) ...     Please Check
PLAT055_ALERT_1_C Maximum Crystal Dimension Missing (or Error) ...     Please Check
PLAT084_ALERT_3_C High wR2 Value (i.e. > 0.25) ..................       0.27 Report
PLAT220_ALERT_2_C NonSolvent   Resd 1  C   Ueq(max)/Ueq(min) Range       4.5 Ratio
PLAT222_ALERT_3_C NonSolvent Resd 1  H   Uiso(max)/Uiso(min) Range       5.1 Ratio
PLAT242_ALERT_2_C Low    ’MainMol’ Ueq as Compared to Neighbors of       C11 Check
PLAT340_ALERT_3_C Low Bond Precision on  C-C Bonds ..............    0.00467 Ang.
```

Alert level G

```
PLAT002_ALERT_2_G Number of Distance or Angle Restraints on AtSite          5 Note
PLAT003_ALERT_2_G Number of Uiso or Uij Restrained non-H Atoms ...          5 Report
PLAT005_ALERT_5_G No Embedded Refinement Details Found  in the CIF     Please Do !
PLAT007_ALERT_5_G Number of Unrefined Donor-H Atoms .............           2 Report
PLAT072_ALERT_2_G SHELXL First  Parameter in WGHT  Unusually Large       0.13 Report
PLAT093_ALERT_1_G No s.u.’s on H-positions, Refinement Reported as      mixed Check
PLAT154_ALERT_1_G The s.u.’s on the Cell Angles are Equal ..(Note)      0.006 Degree
PLAT199_ALERT_1_G Reported _cell_measurement_temperature ..... (K)        273 Check
PLAT200_ALERT_1_G Reported   _diffrn_ambient_temperature ..... (K)        273 Check
PLAT230_ALERT_2_G Hirshfeld Test Diff for    C21      --C22      .        5.9 s.u.
PLAT301_ALERT_3_G Main Residue  Disorder ..............(Resd  1  )         7% Note
PLAT410_ALERT_2_G Short Intra H...H Contact  H16      ..H20C     .       1.90 Ang.
                                                  x,y,z  =      1_555 Check
PLAT720_ALERT_4_G Number of Unusual/Non-Standard Labels .........           5 Note
PLAT779_ALERT_4_G Suspect or Irrelevant (Bond) Angle(s) in CIF ...      36.90 Deg.
               H20A -C20  -H20D    1_555   1_555   1_555 ......     #    3 Check
PLAT779_ALERT_4_G Suspect or Irrelevant (Bond) Angle(s) in CIF ...      43.00 Deg.
               H20B -C20  -H20C    1_555   1_555   1_555 ......     #    4 Check
PLAT779_ALERT_4_G Suspect or Irrelevant (Bond) Angle(s) in CIF ...      41.70 Deg.
               C1B  -C20  -C21     1_555   1_555   1_555 ......     #   21 Check
PLAT860_ALERT_3_G Number of Least-Squares Restraints ............          60 Note
```

0 **ALERT level A** = Most likely a serious problem - resolve or explain
0 **ALERT level B** = A potentially serious problem, consider carefully
9 **ALERT level C** = Check. Ensure it is not caused by an omission or oversight
17 **ALERT level G** = General information/check it is not something unexpected

7 ALERT type 1 CIF construction/syntax error, inconsistent or missing data
7 ALERT type 2 Indicator that the structure model may be wrong or deficient
6 ALERT type 3 Indicator that the structure quality may be low
4 ALERT type 4 Improvement, methodology, query or suggestion
2 ALERT type 5 Informative message, check

It is advisable to attempt to resolve as many as possible of the alerts in all categories. Often the minor alerts point to easily fixed oversights, errors and omissions in your CIF or refinement strategy, so attention to these fine details can be worthwhile. In order to resolve some of the more serious problems it may be necessary to carry out additional measurements or structure refinements. However, the purpose of your study may justify the reported deviations and the more serious of these should normally be commented upon in the discussion or experimental section of a paper or in the "special_details" fields of the CIF. checkCIF was carefully designed to identify outliers and unusual parameters, but every test has its limitations and alerts that are not important in a particular case may appear. Conversely, the absence of alerts does not guarantee there are no aspects of the results needing attention. It is up to the individual to critically assess their own results and, if necessary, seek expert advice.

Publication of your CIF in IUCr journals

A basic structural check has been run on your CIF. These basic checks will be run on all CIFs submitted for publication in IUCr journals (*Acta Crystallographica*, *Journal of Applied Crystallography*, *Journal of Synchrotron Radiation*); however, if you intend to submit to *Acta Crystallographica Section C* or *E* or *IUCrData*, you should make sure that full publication checks are run on the final version of your CIF prior to submission.

Publication of your CIF in other journals

Please refer to the *Notes for Authors* of the relevant journal for any special instructions relating to CIF submission.

PLATON version of 28/11/2022; check.def file version of 28/11/2022

Datablock a - ellipsoid plot

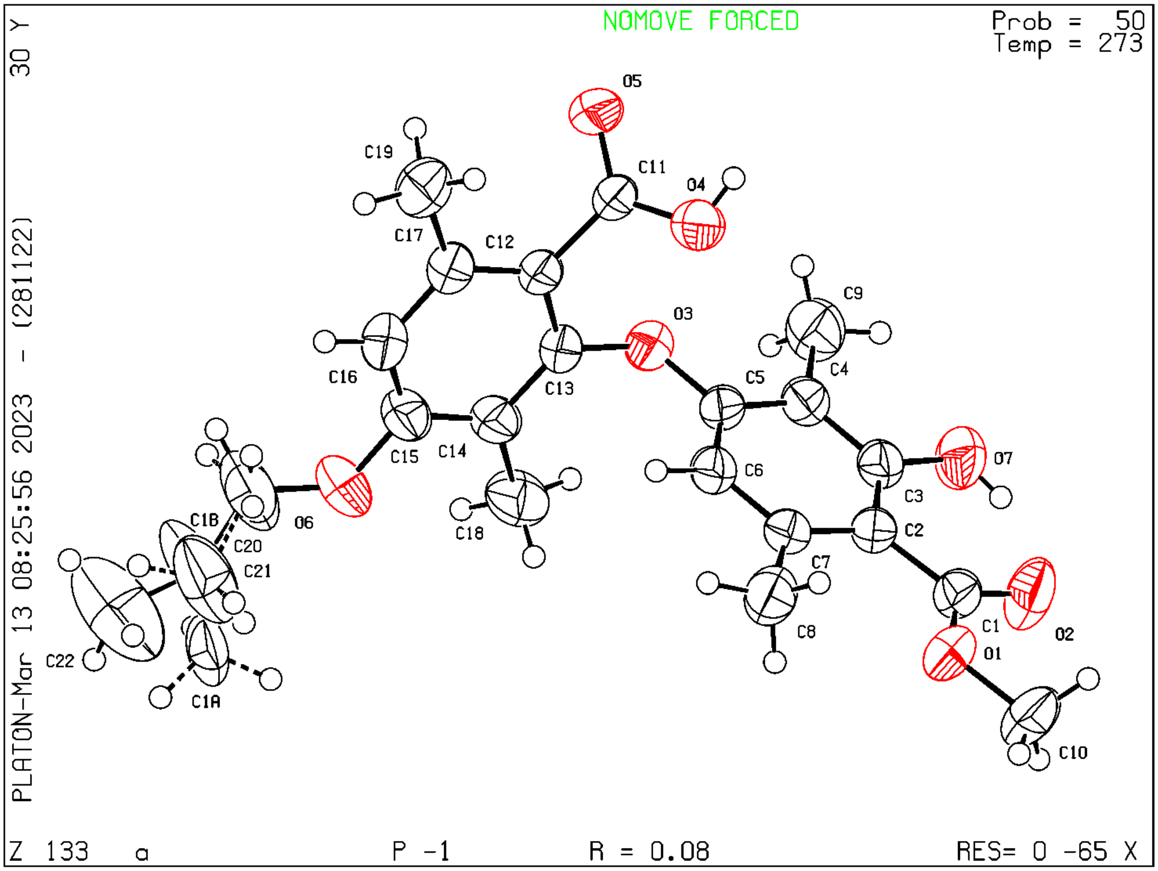

Supplement: Supplementary file 1 [file molecules-28-04303-s001.zip › checkcif (3d).pdf]
