# Supplementary material for: Hydroxide-Mediated SNAr Rearrangement for Synthesis of Novel Depside Derivatives Containing Diaryl Ether Skeleton as Antitumor Agents
Source: Molecules. 2023 May 24;28(11):4303. doi: 10.3390/molecules28114303 (PMC10254537; doi:10.3390/molecules28114303)
Supplement: Supplementary file 1 [file molecules-28-04303-s001.zip › supporting information.pdf]

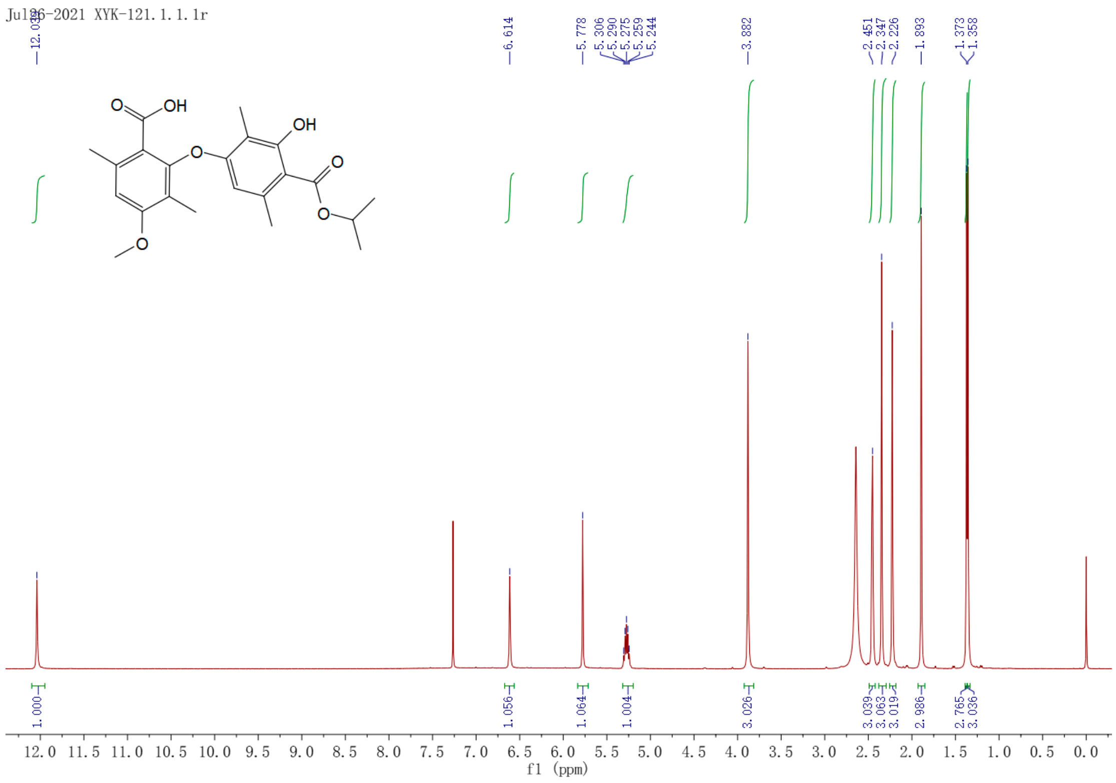

Figure S1. ^{1}H NMR spectrum of compound **3a**

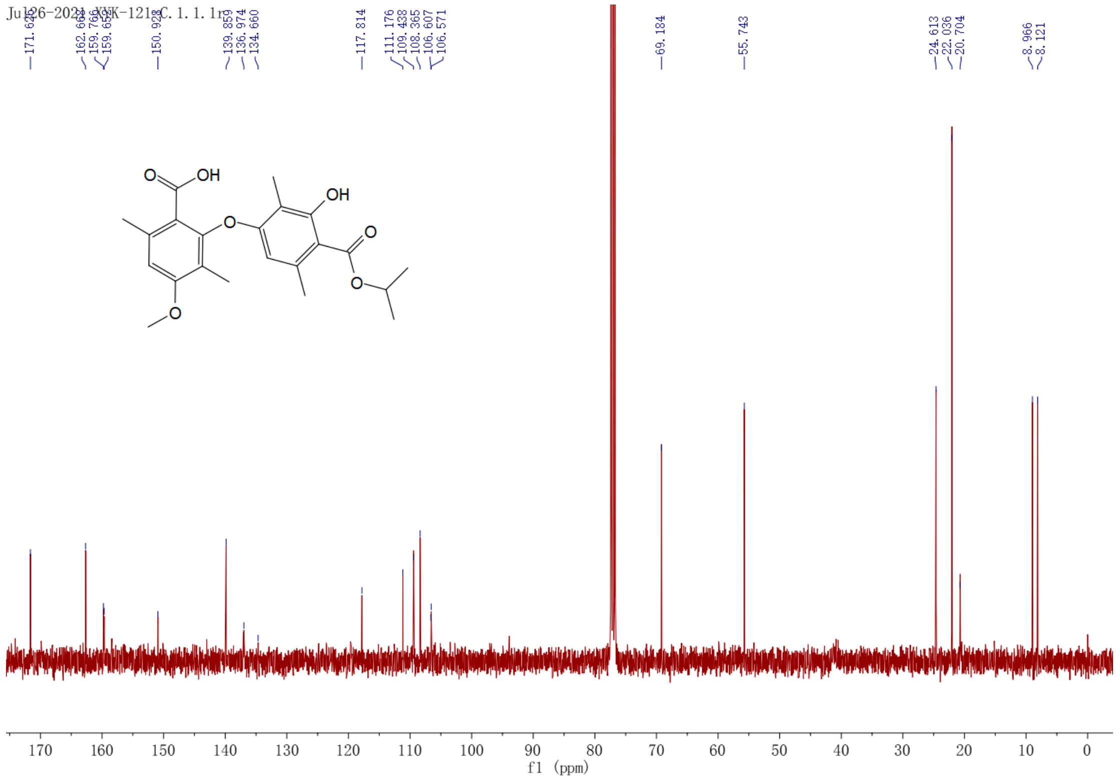

Figure S2. ^{13}C NMR spectrum of compound **3a**

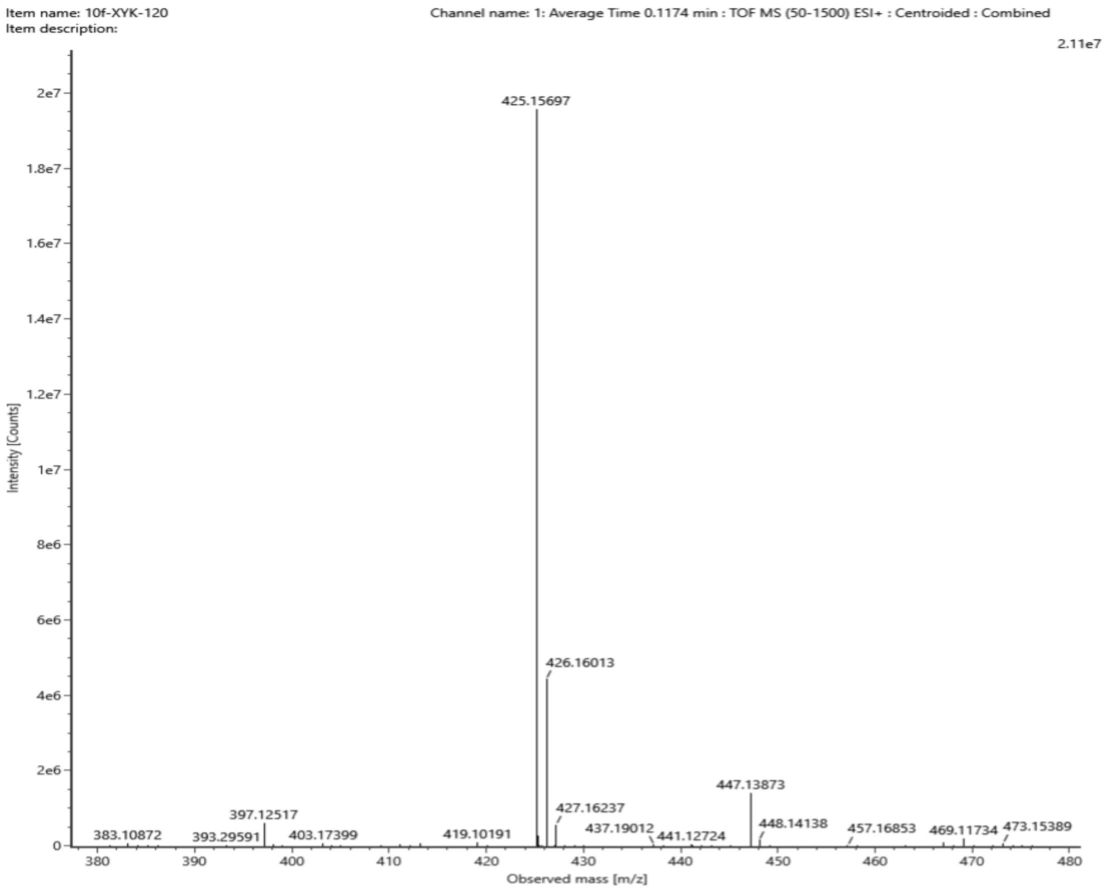

Figure S3. HRMS spectrum of compound **3a**

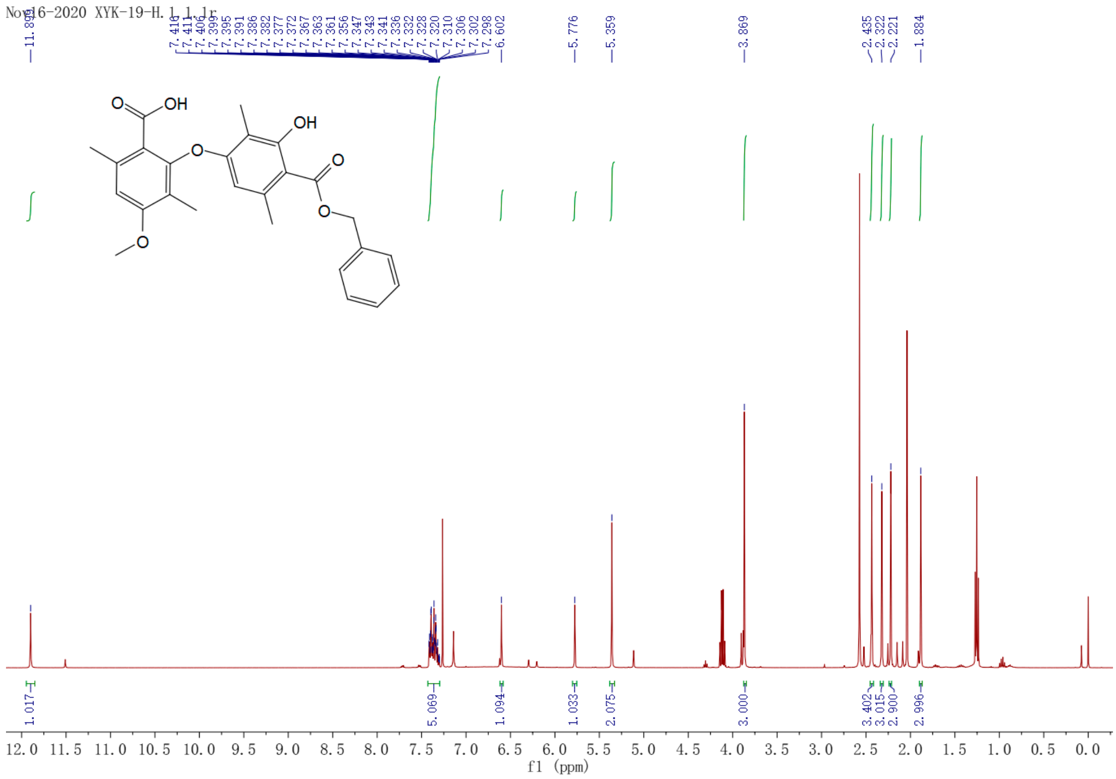

Figure S4. ^{1}H NMR spectrum of compound **3b**

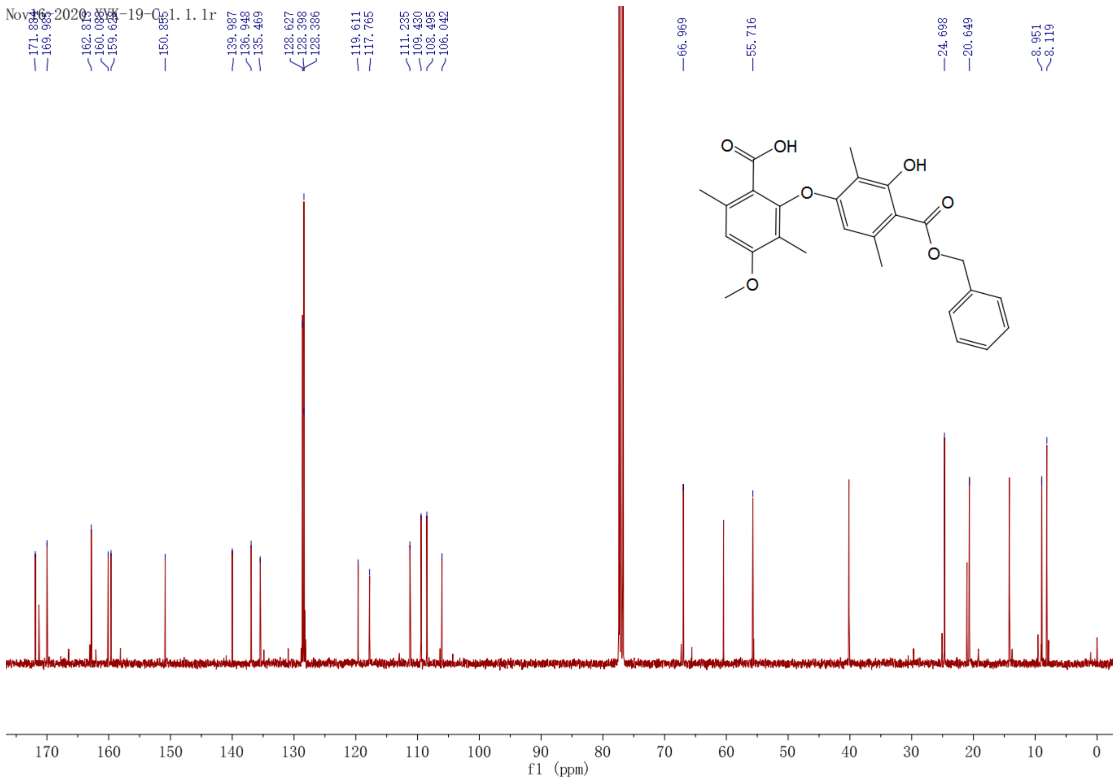

Figure S5. ^{13}C NMR spectrum of compound **3b**

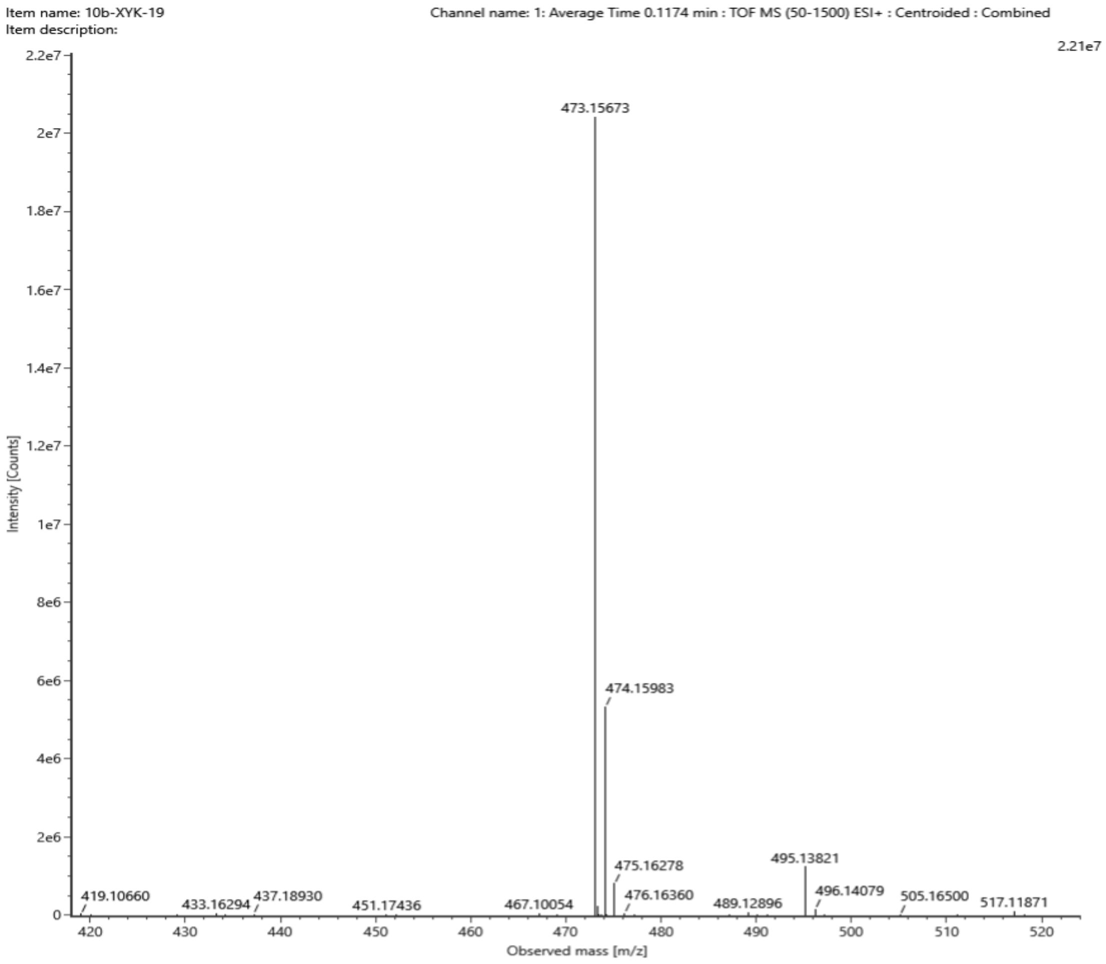

Figure S6. HRMS spectrum of compound **3b**

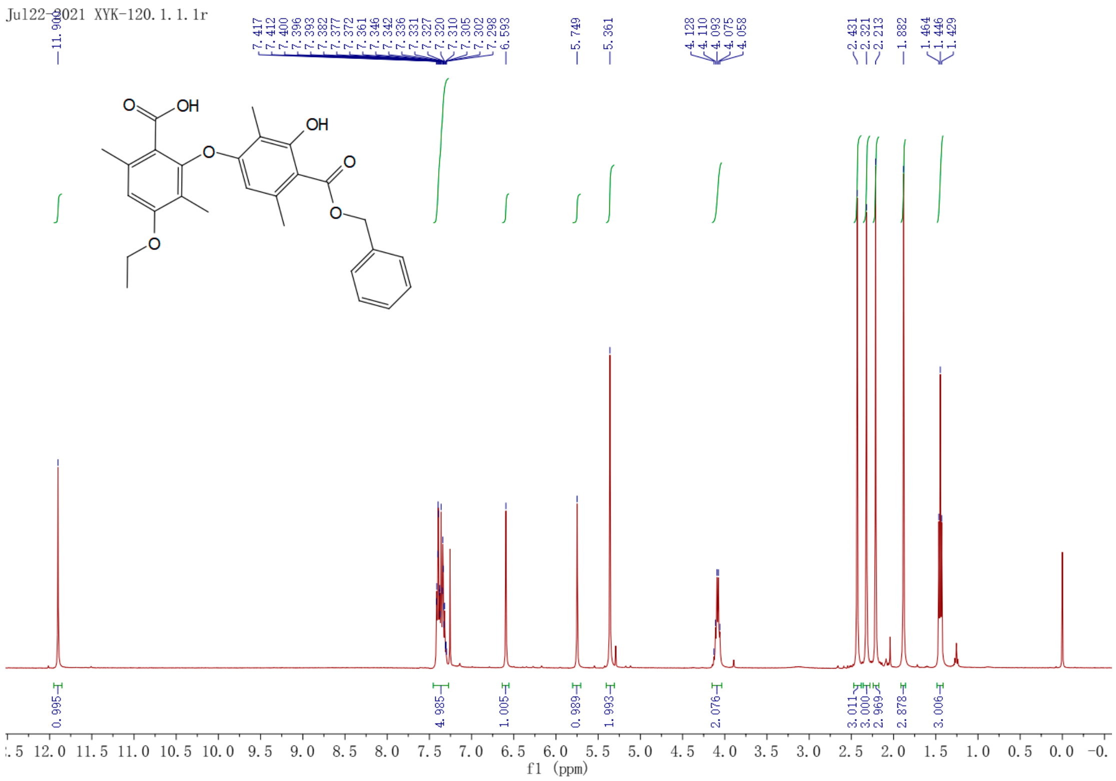

Figure S7. ^{1}H NMR spectrum of compound **3c**

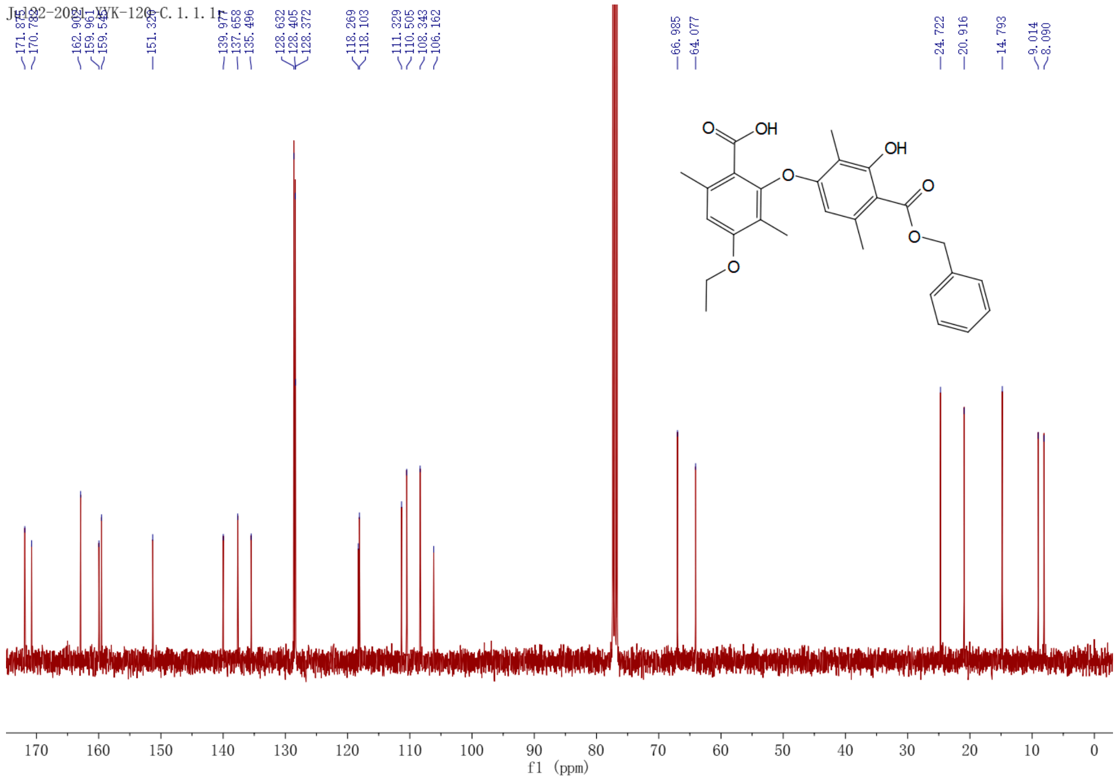

Figure S8. ^{13}C NMR spectrum of compound **3c**

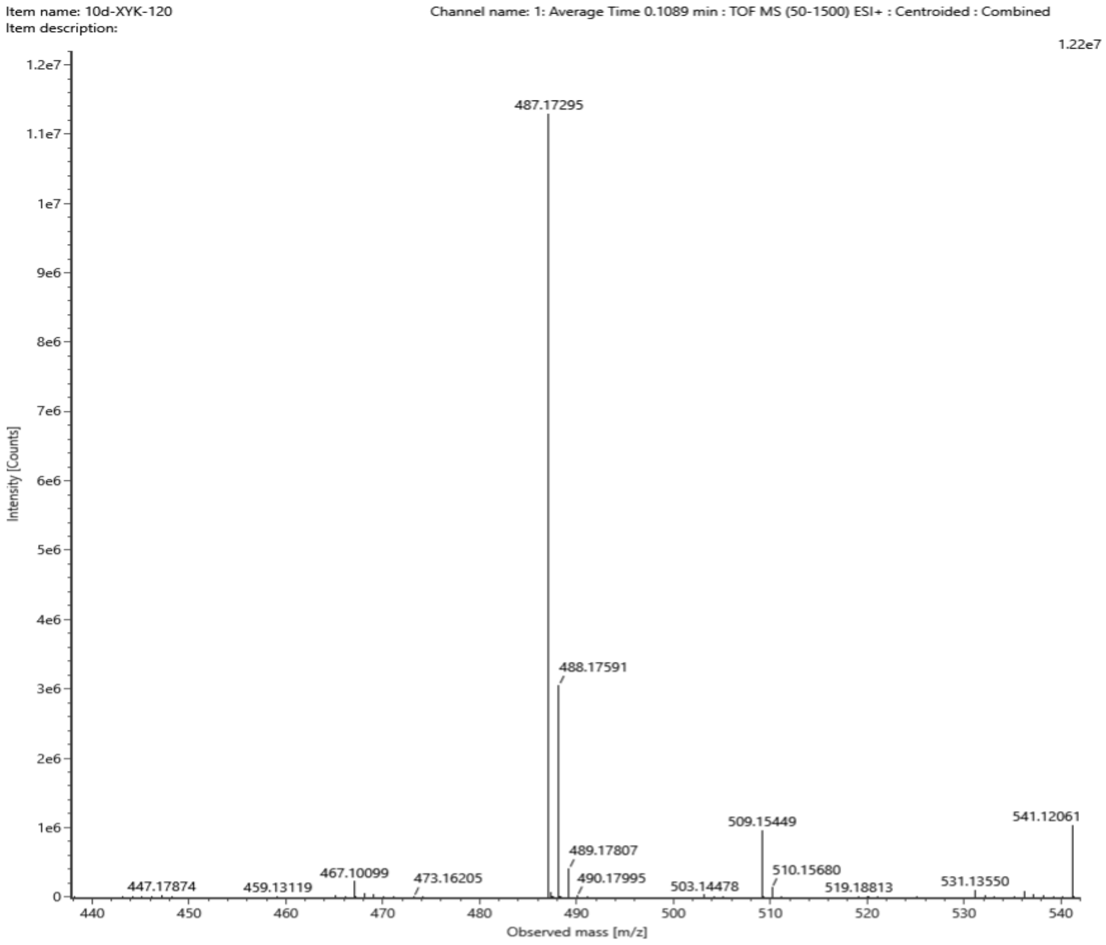

Figure S9. HRMS spectrum of compound **3c**

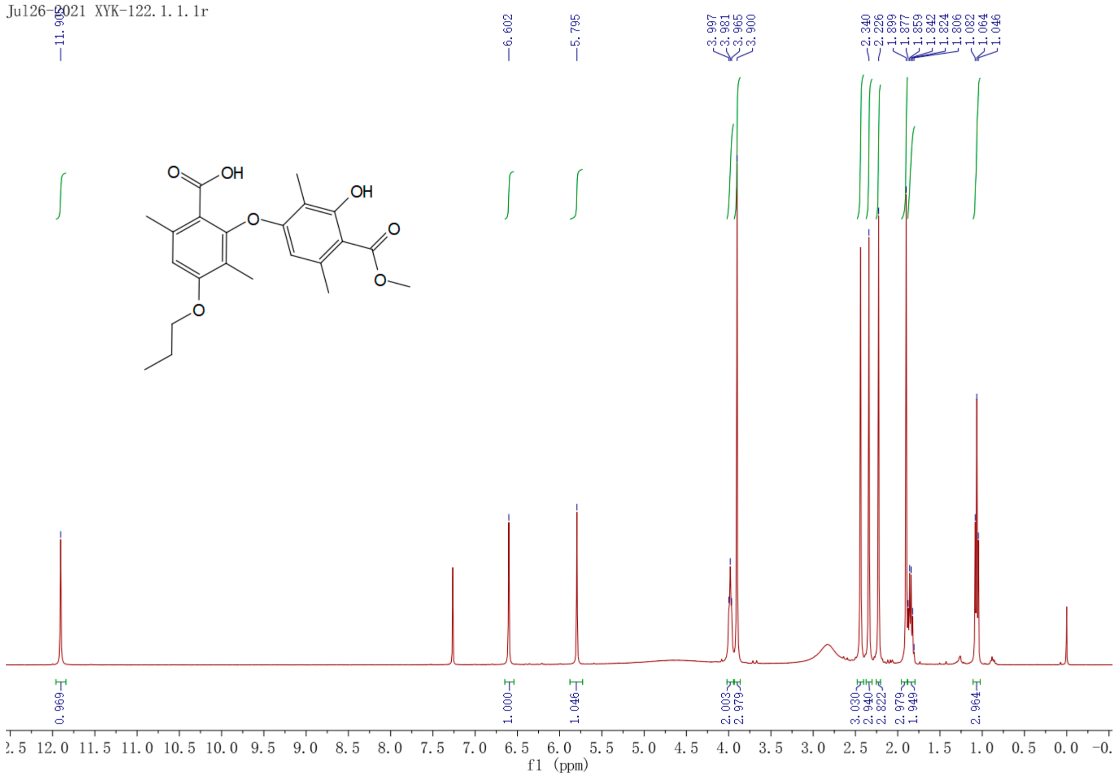

Figure S10. ^{1}H NMR spectrum of compound **3d**

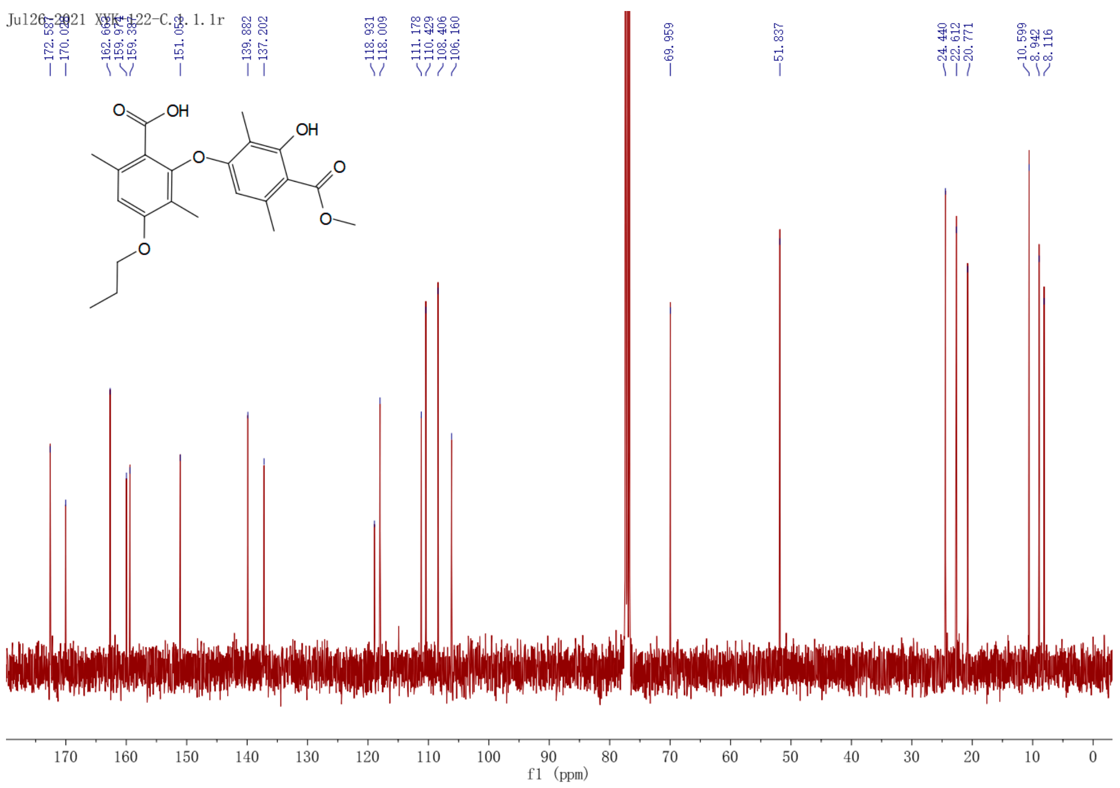

Figure S11. ^{13}C NMR spectrum of compound **3d**

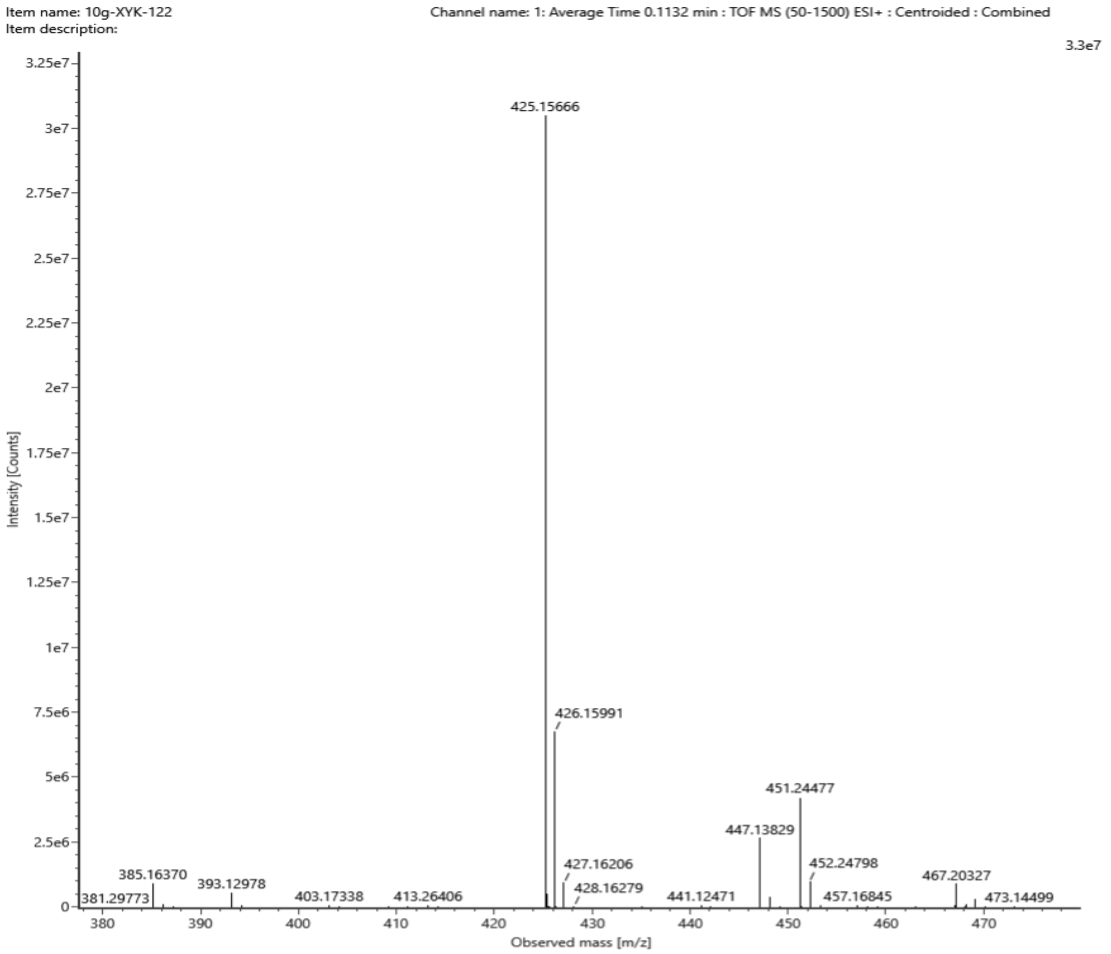

Figure S12. HRMS spectrum of compound **3d**

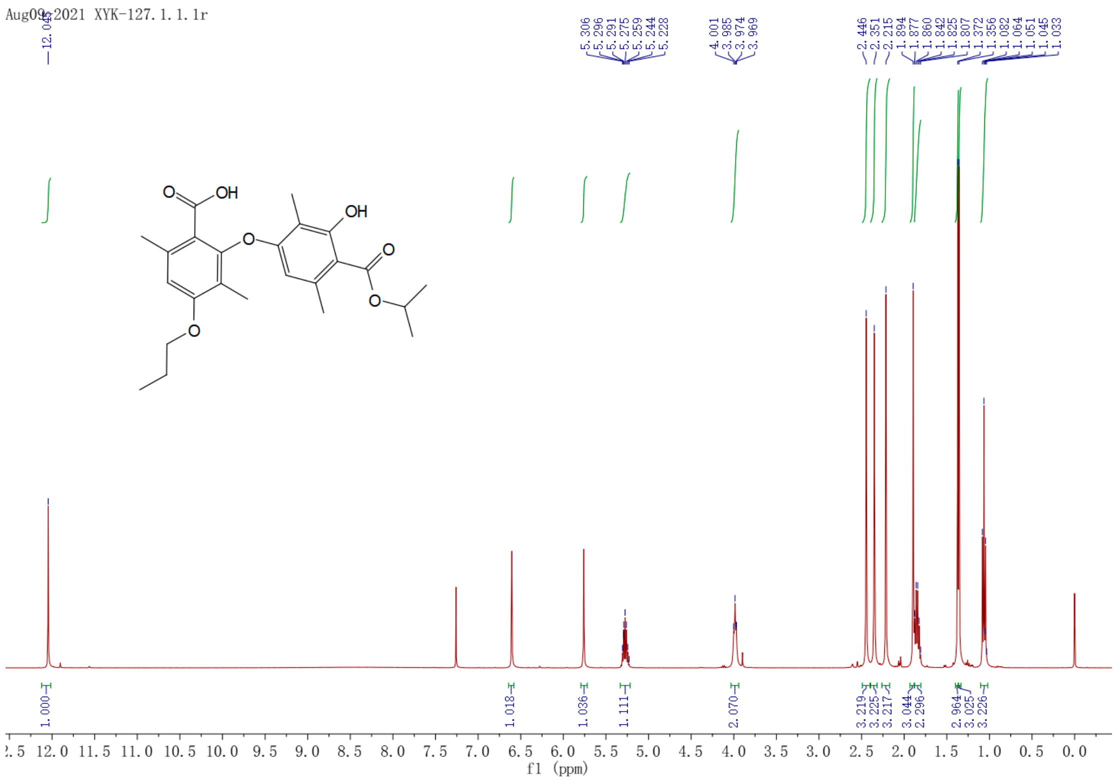

Figure S13. ^{1}H NMR spectrum of compound **3e**

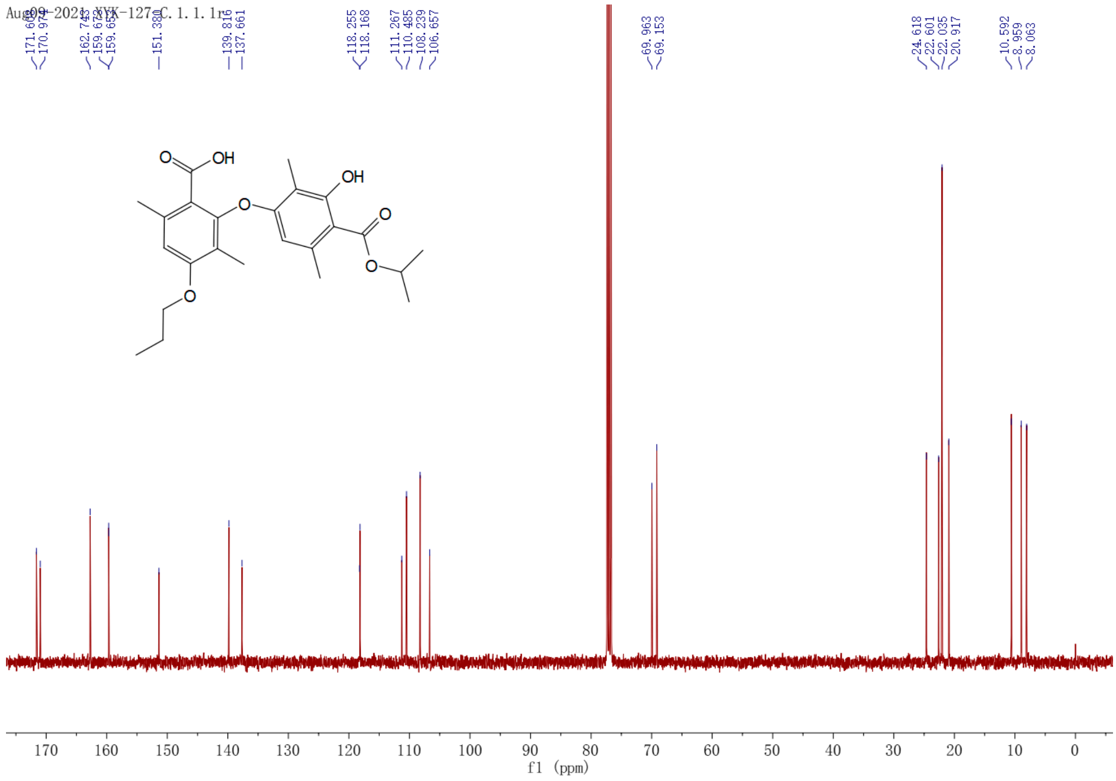

Figure S14. ^{13}C NMR spectrum of compound **3e**

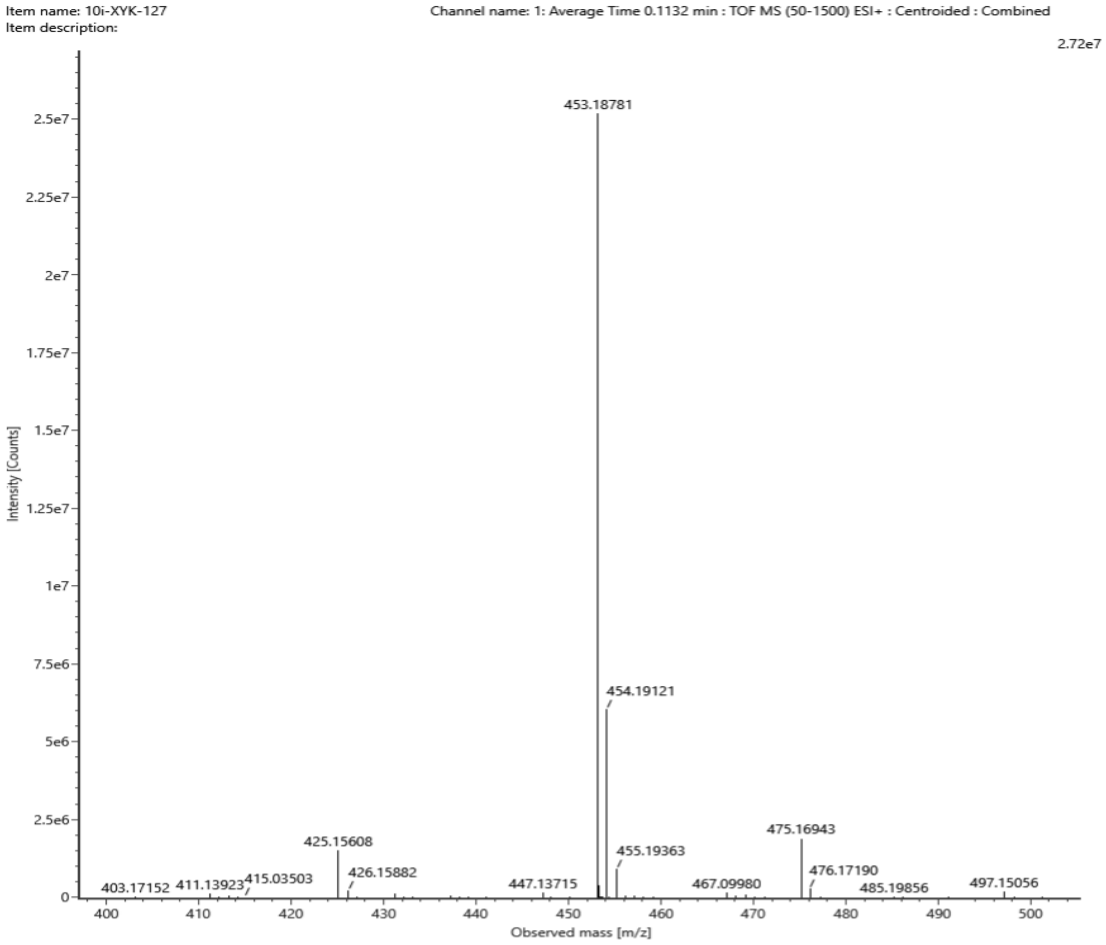

Figure S15. HRMS spectrum of compound **3e**

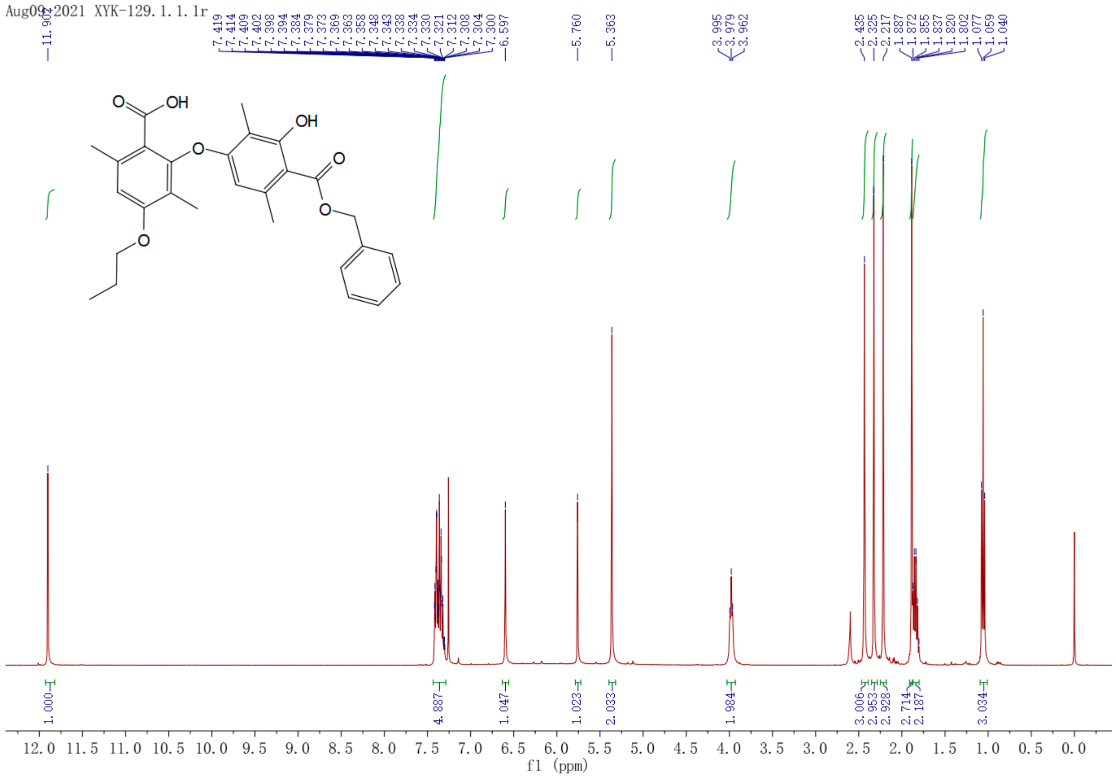

Figure S16. ^{1}H NMR spectrum of compound **3f**

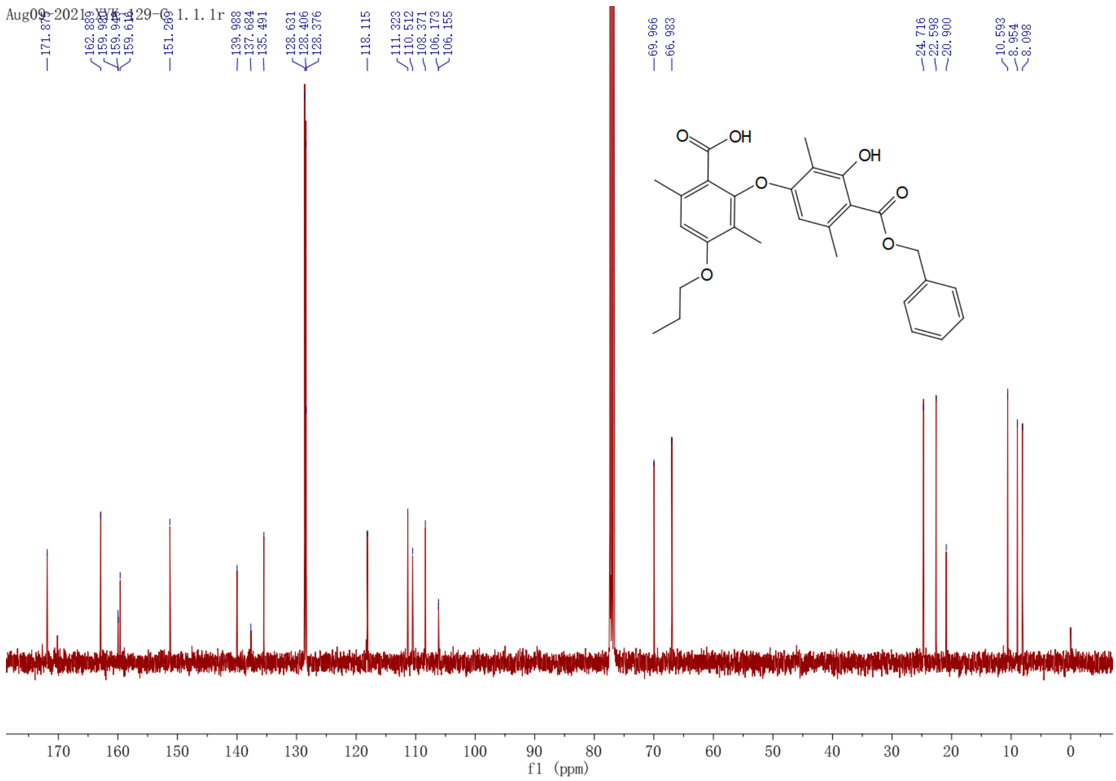

Figure S17. ^{13}C NMR spectrum of compound **3f**

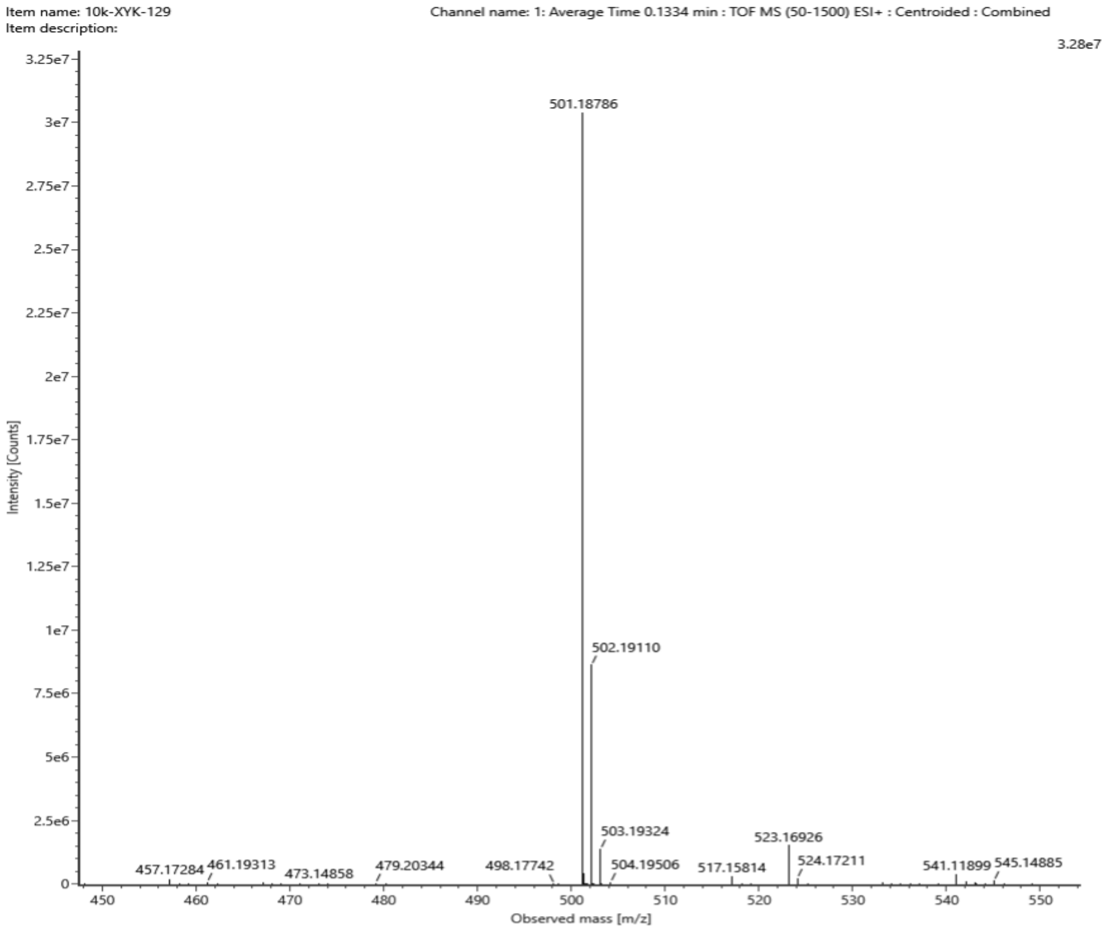

Figure S18. HRMS spectrum of compound **3f**

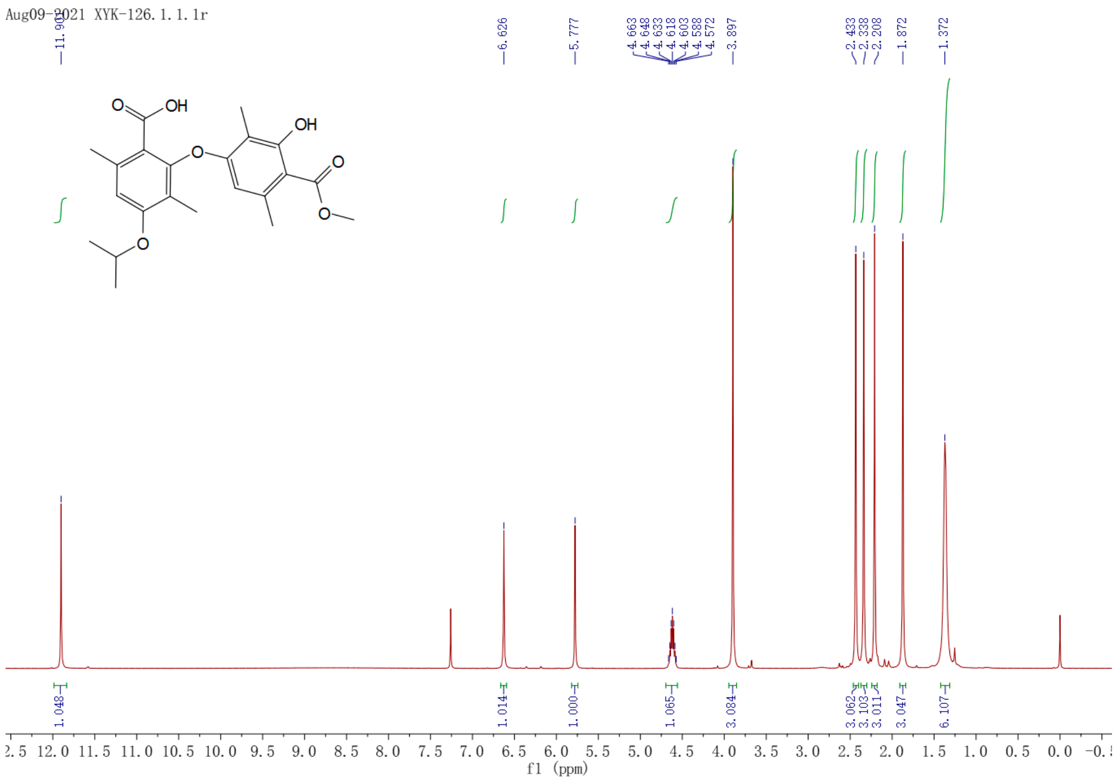

Figure S19. ^{1}H NMR spectrum of compound **3g**

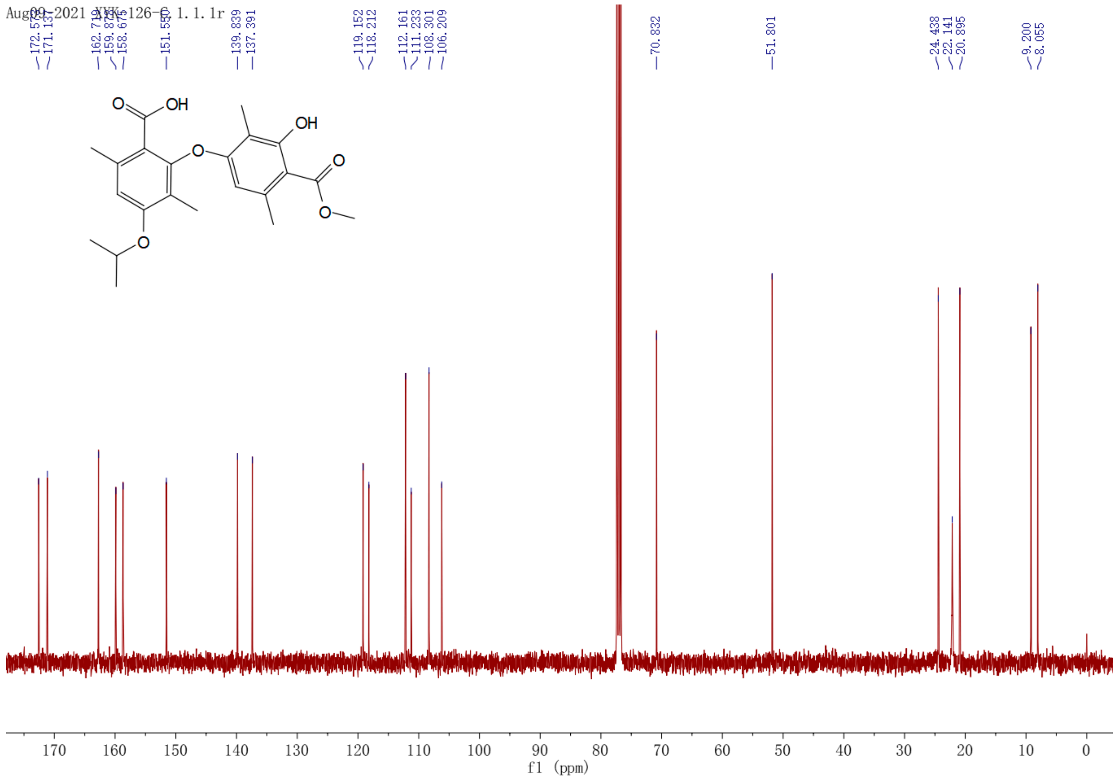

Figure S20. ^{13}C NMR spectrum of compound **3g**

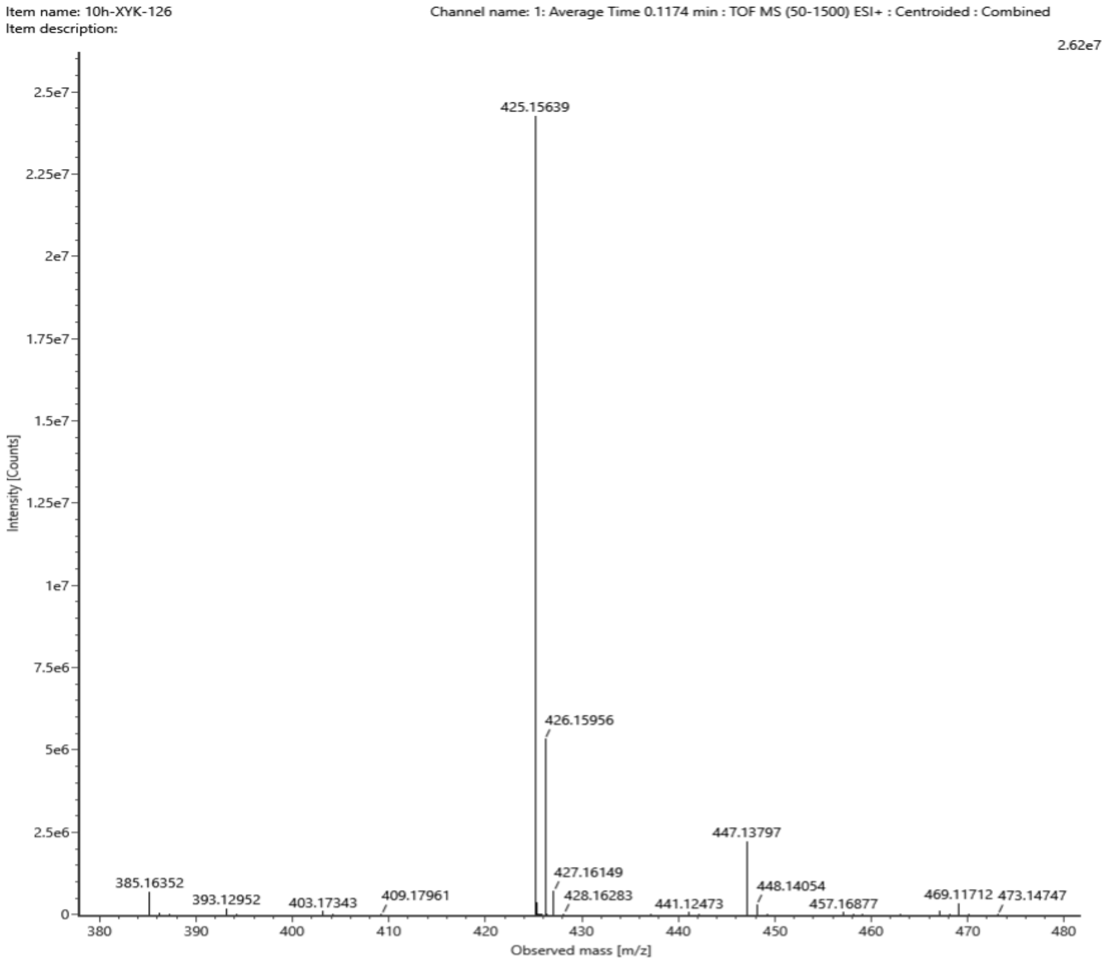

Figure S21. HRMS spectrum of compound **3g**

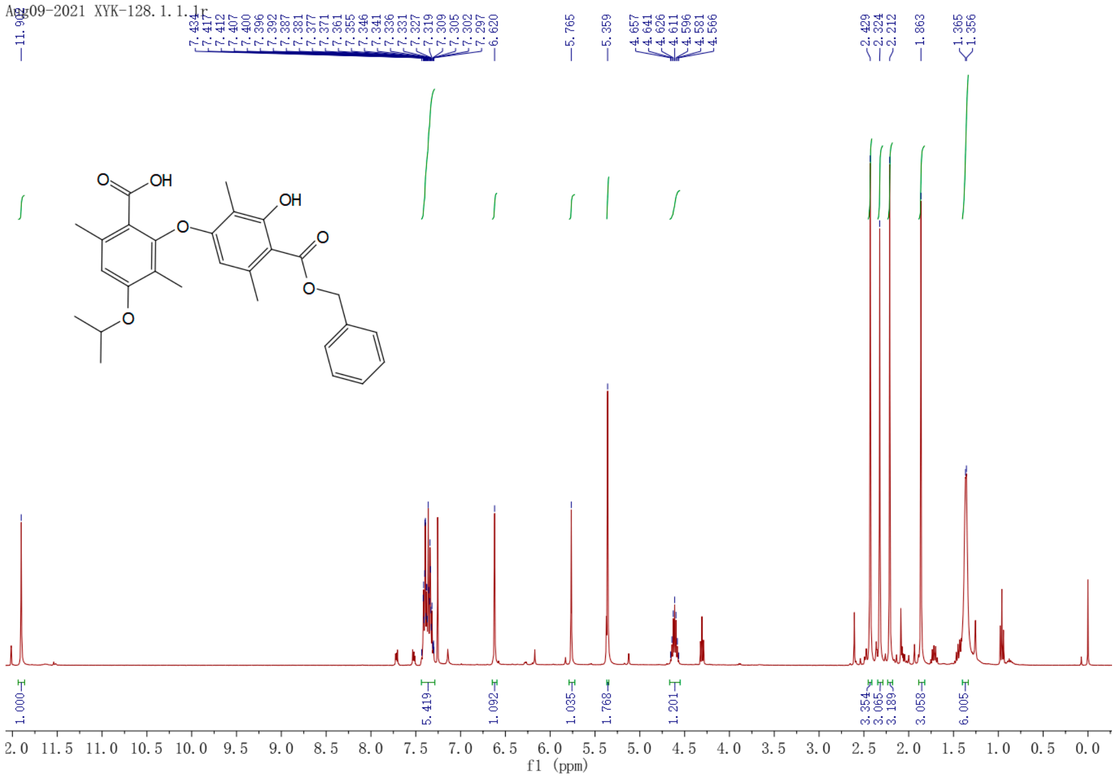

Figure S22. ^{1}H NMR spectrum of compound **3h**

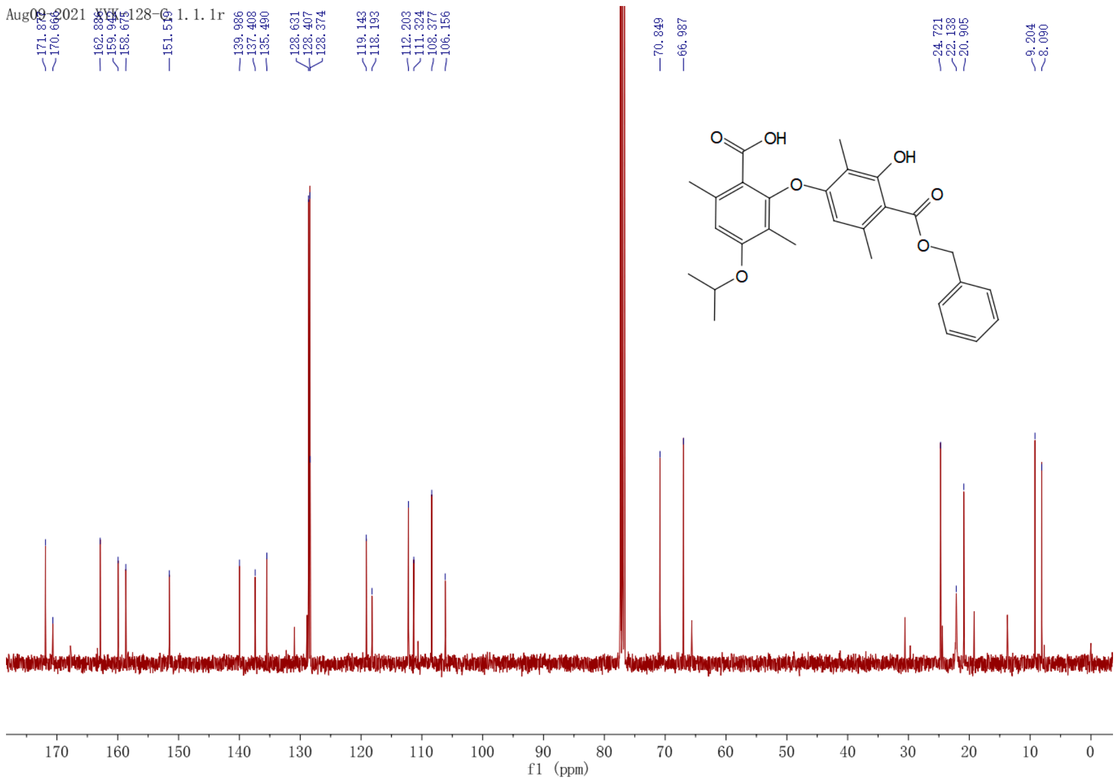

Figure S23. ^{13}C NMR spectrum of compound **3h**

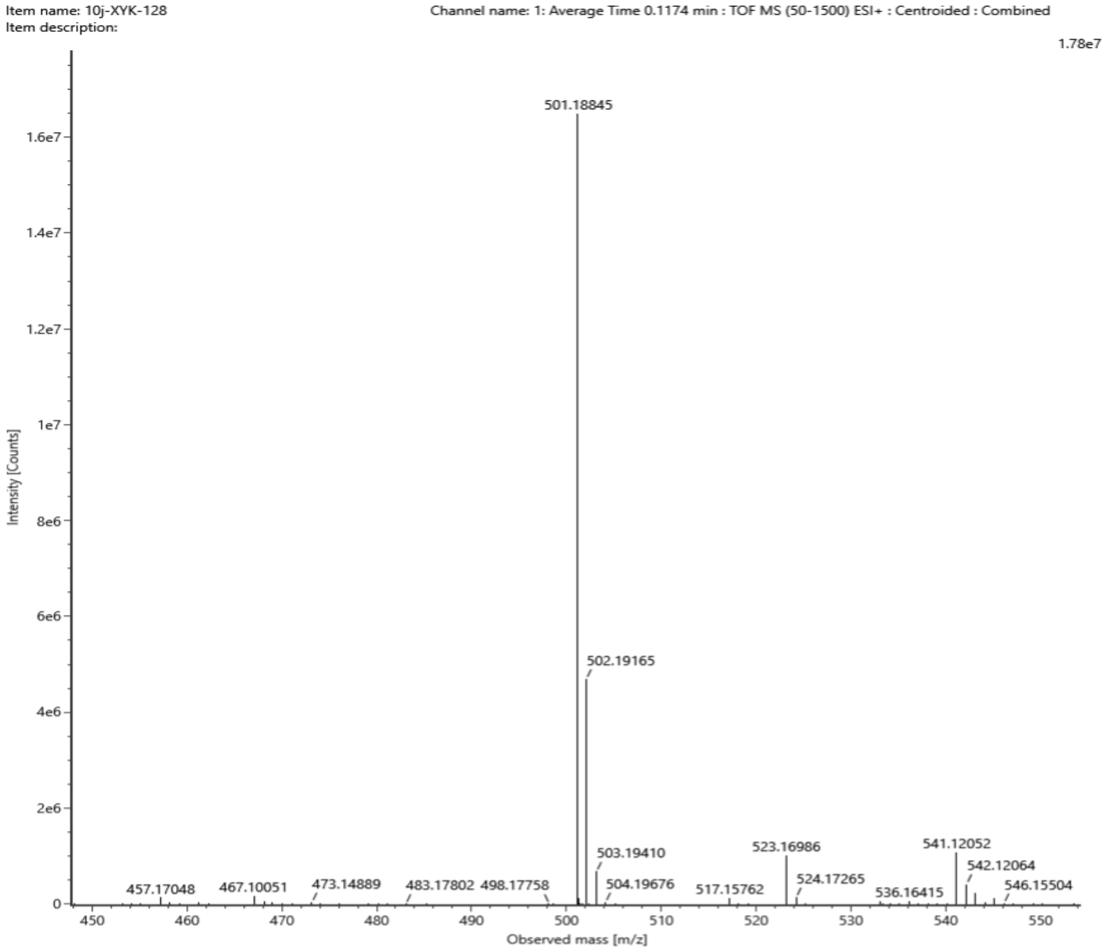

Figure S24. HRMS spectrum of compound **3h**

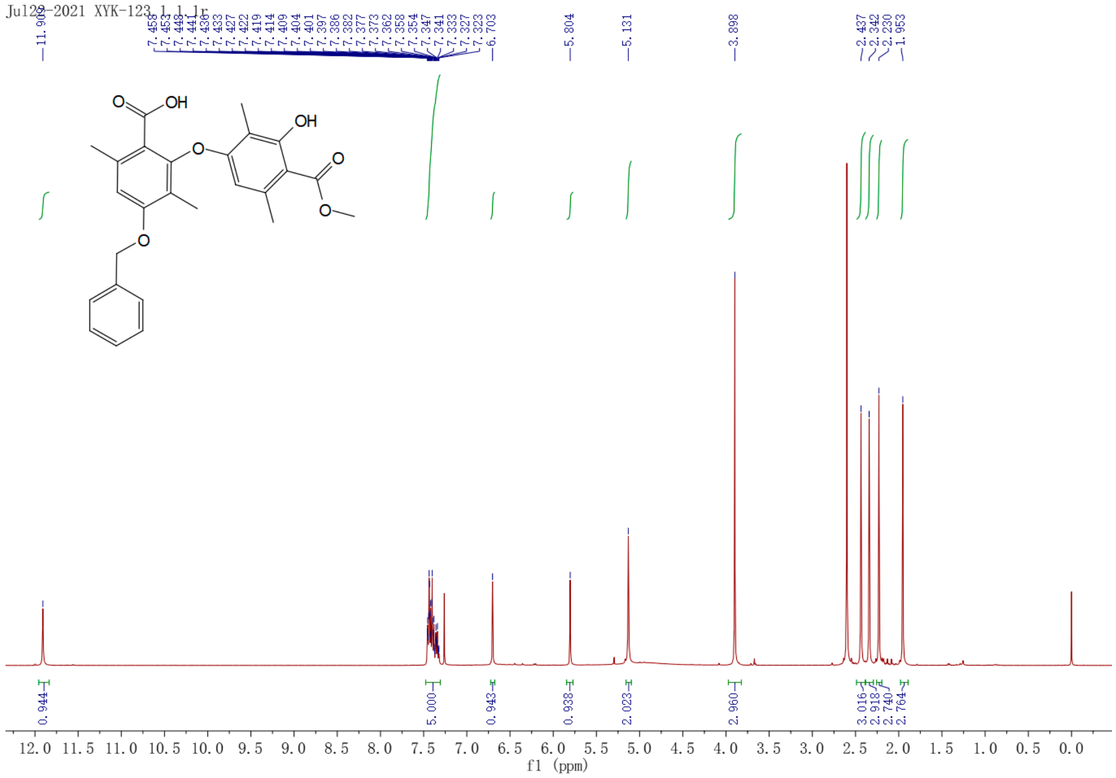

Figure S25. ^{1}H NMR spectrum of compound **3i**

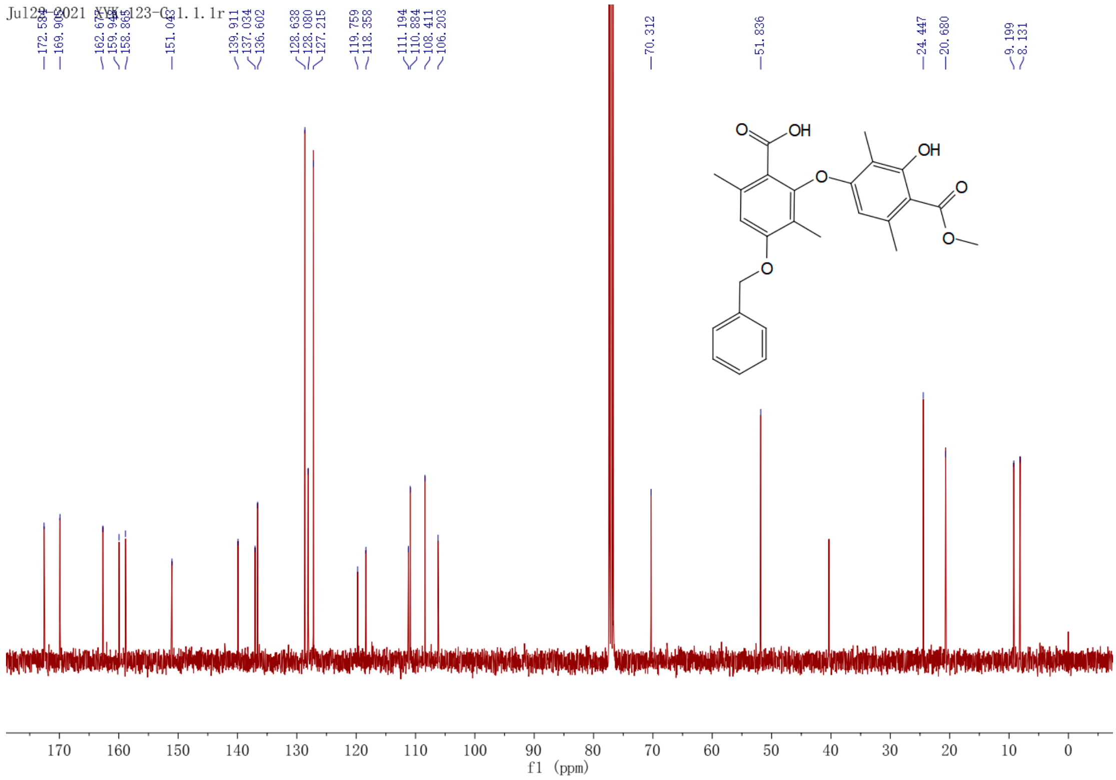

Figure S26. ^{13}C NMR spectrum of compound **3i**

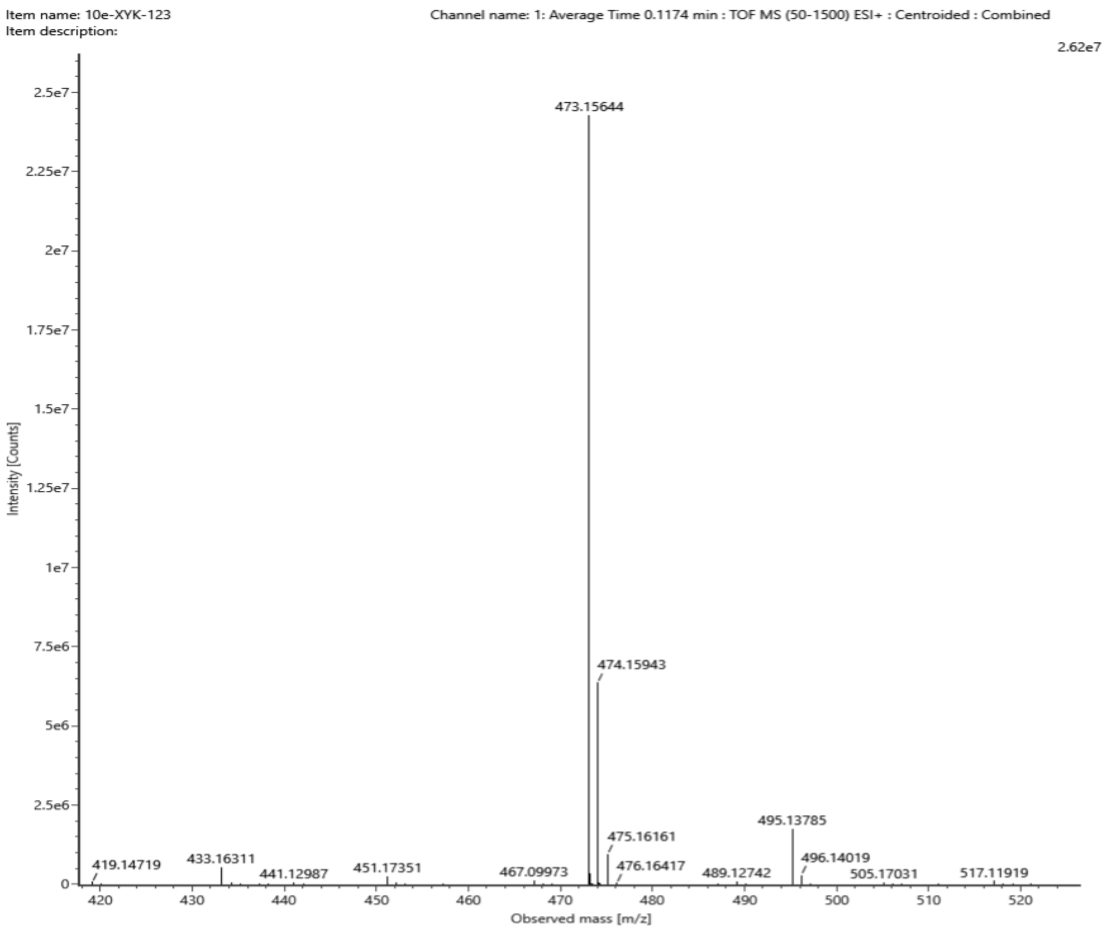

Figure S27. HRMS spectrum of compound **3i**

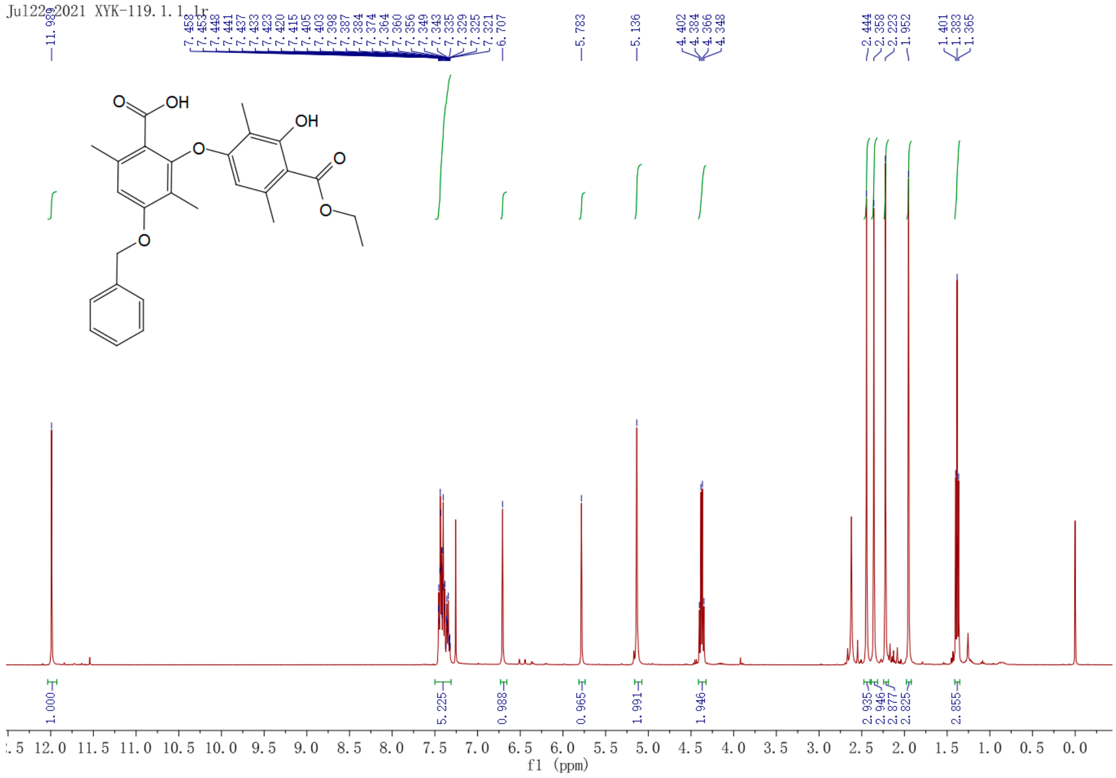

Figure S28. ^{1}H NMR spectrum of compound **3j**

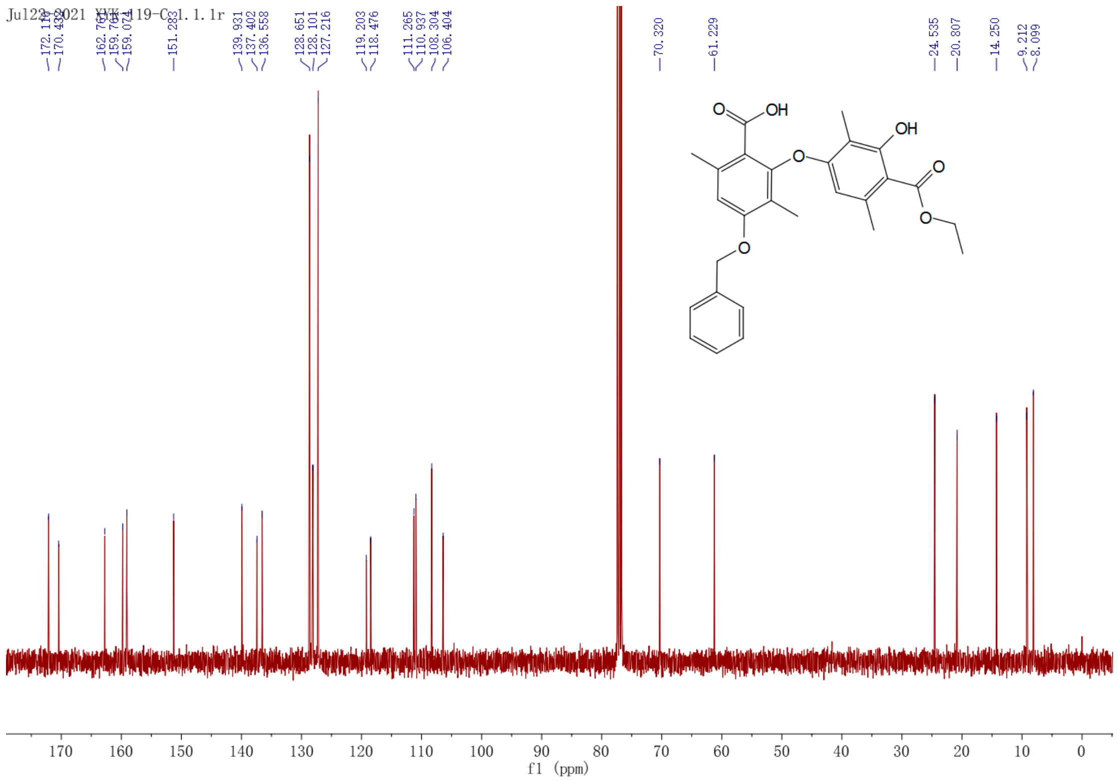

Figure S29. ^{13}C NMR spectrum of compound **3j**

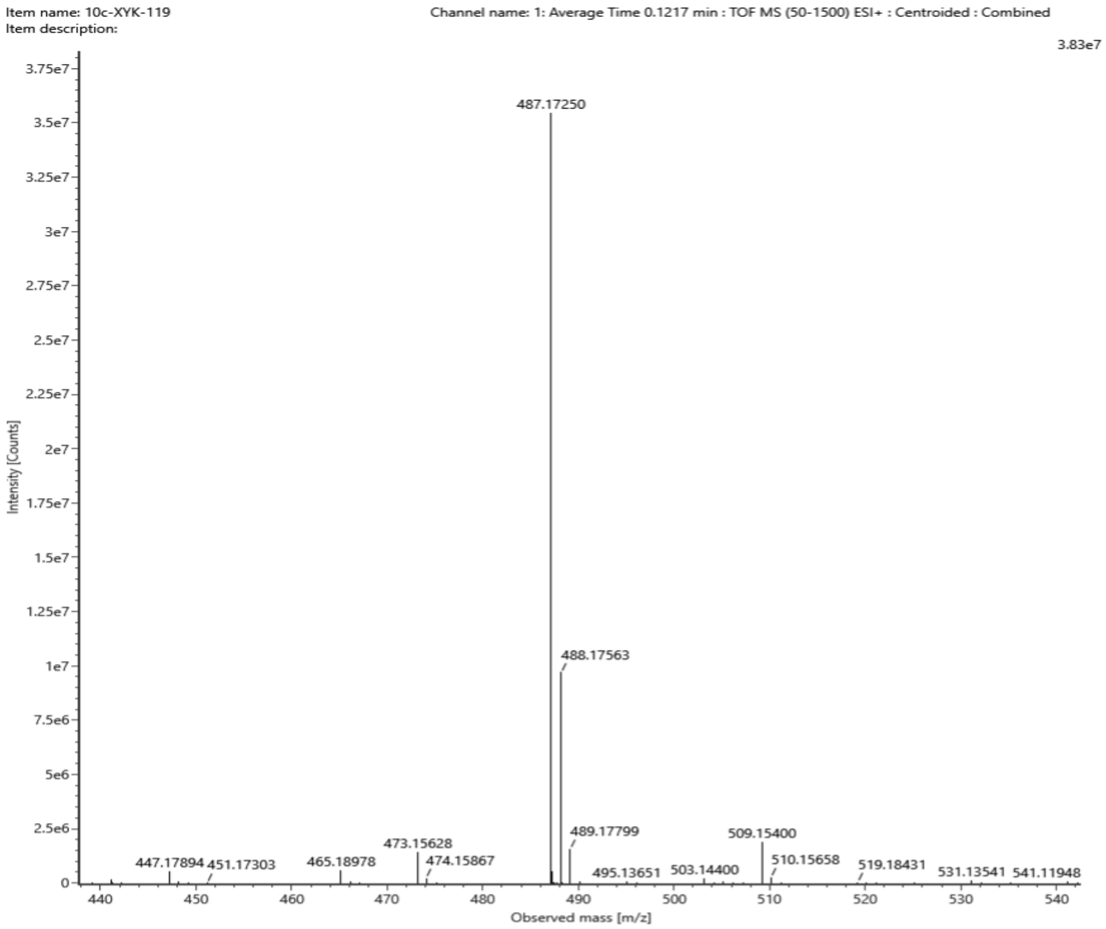

Figure S30. HRMS spectrum of compound **3j**
